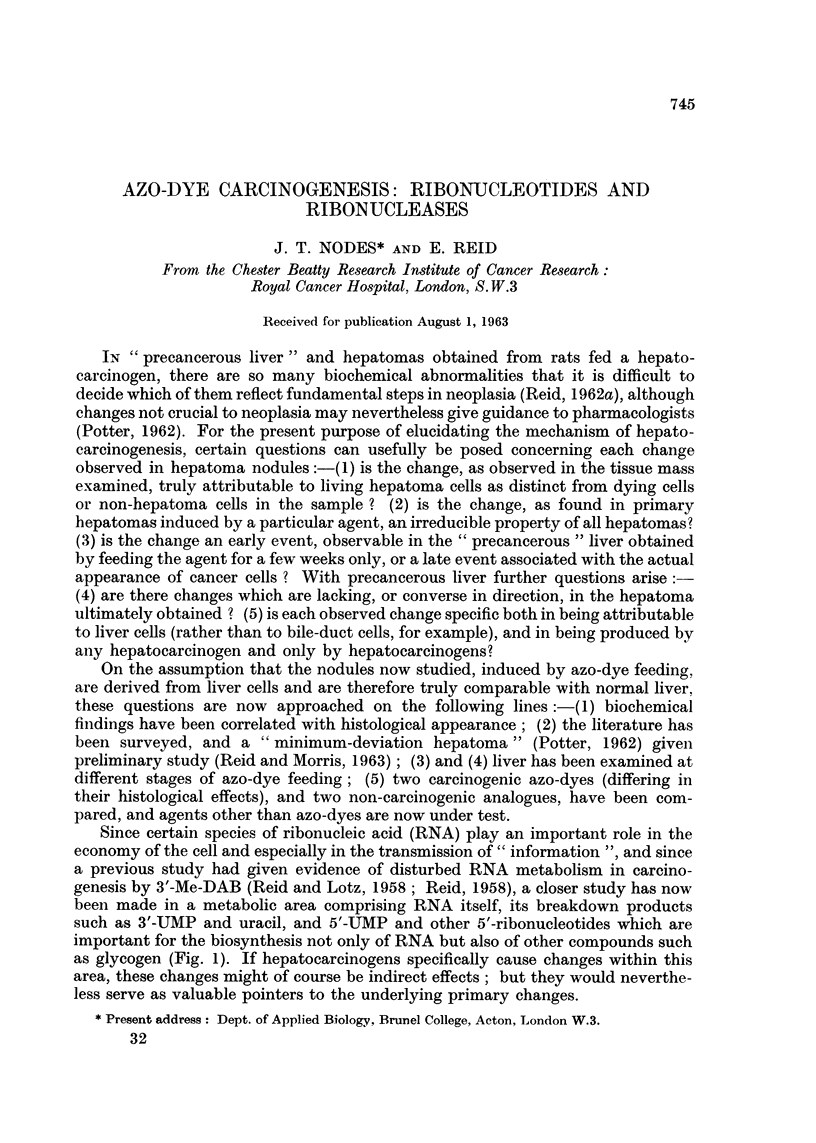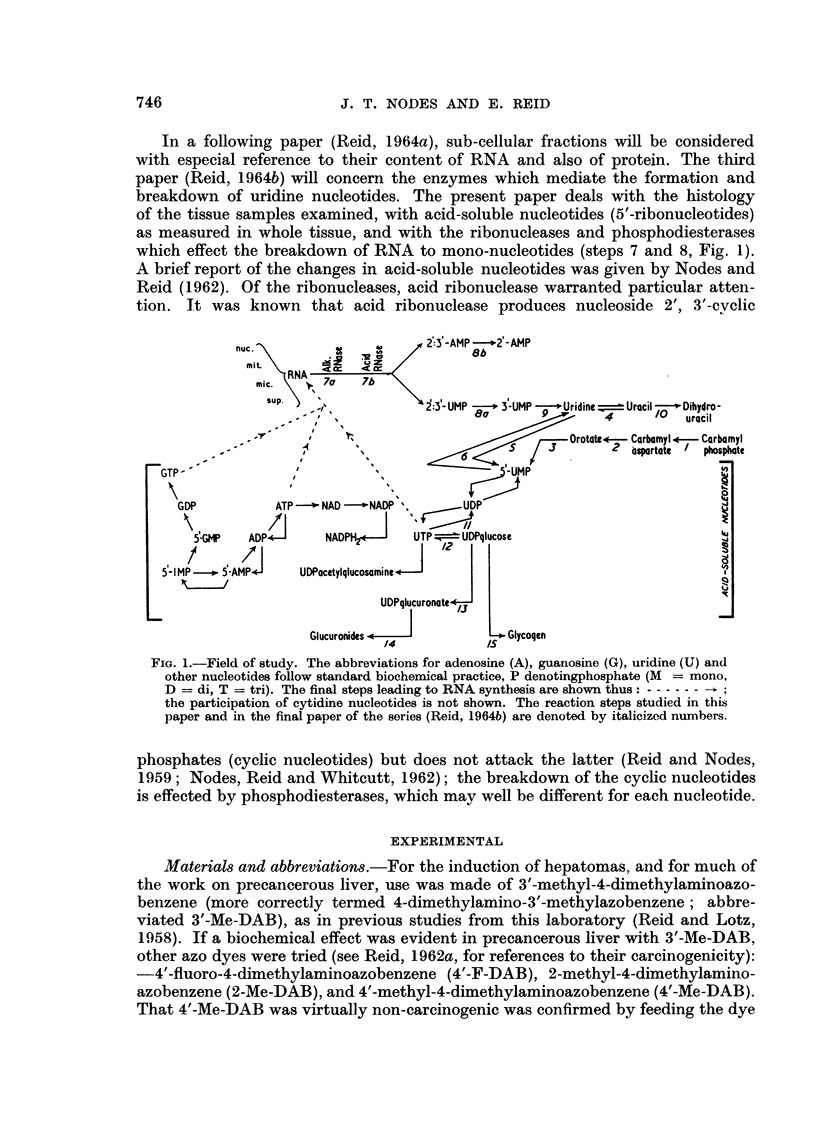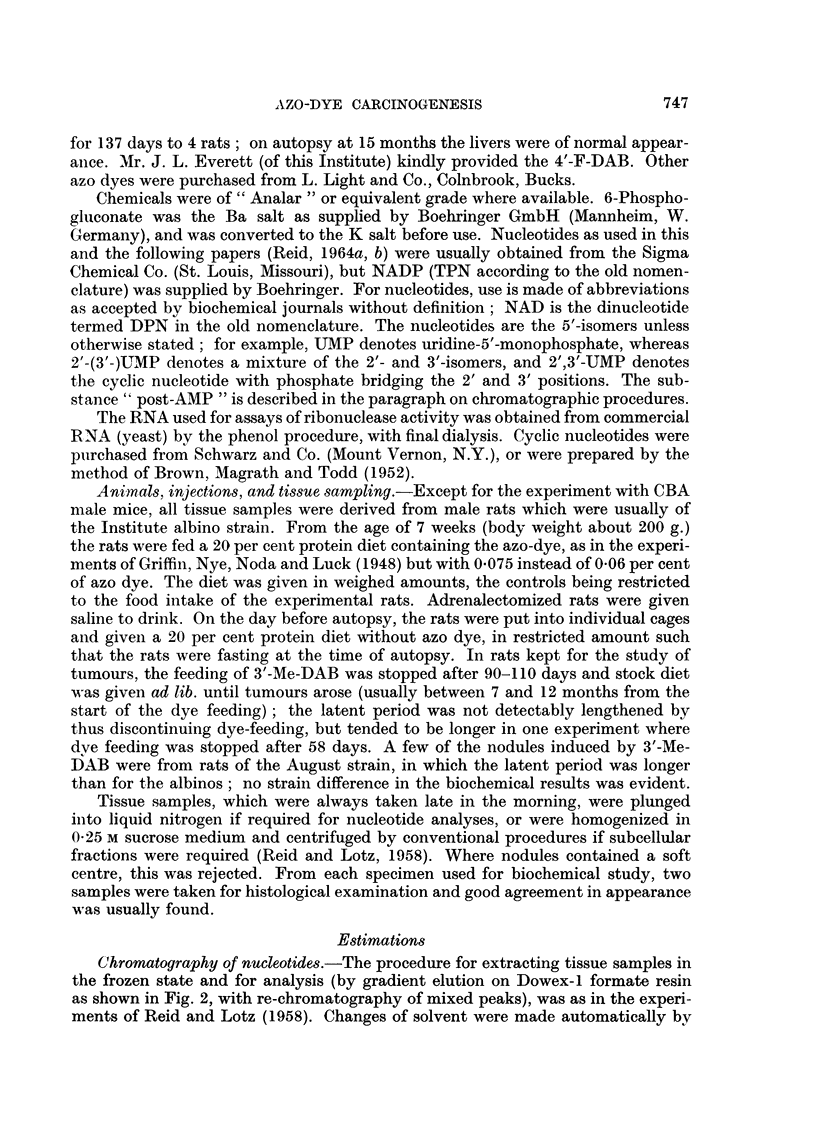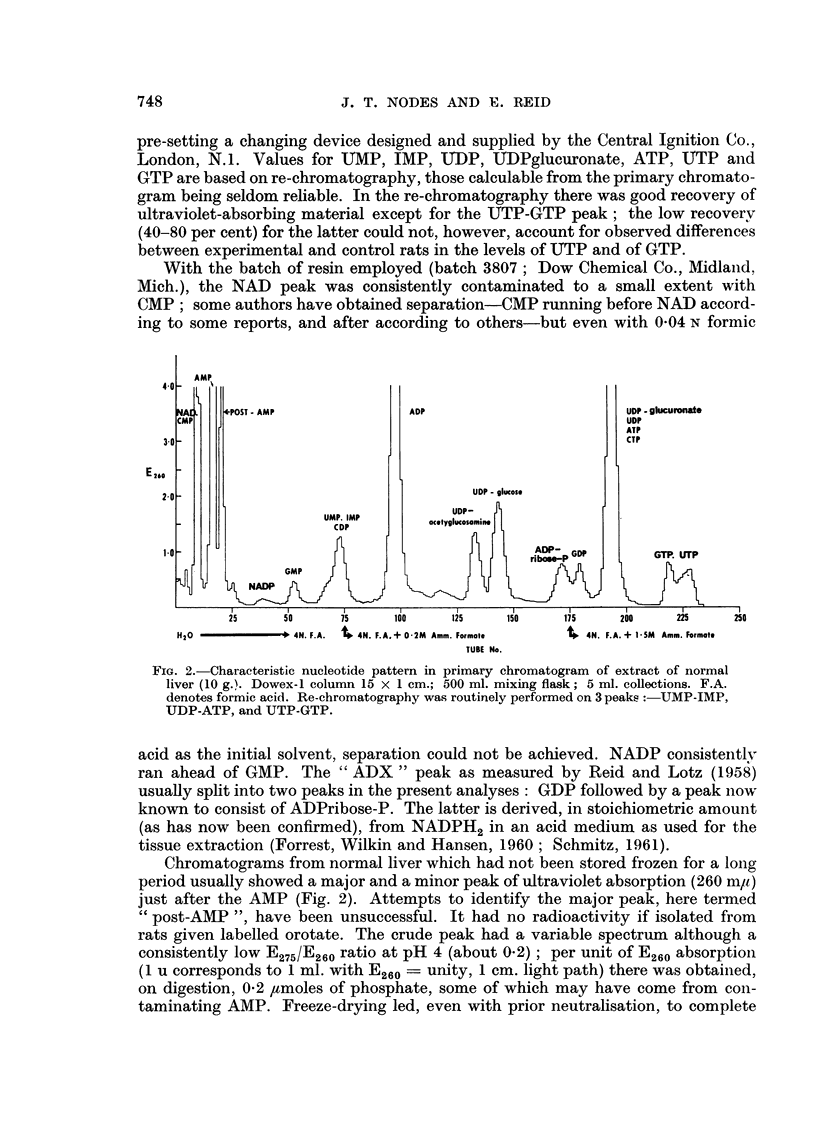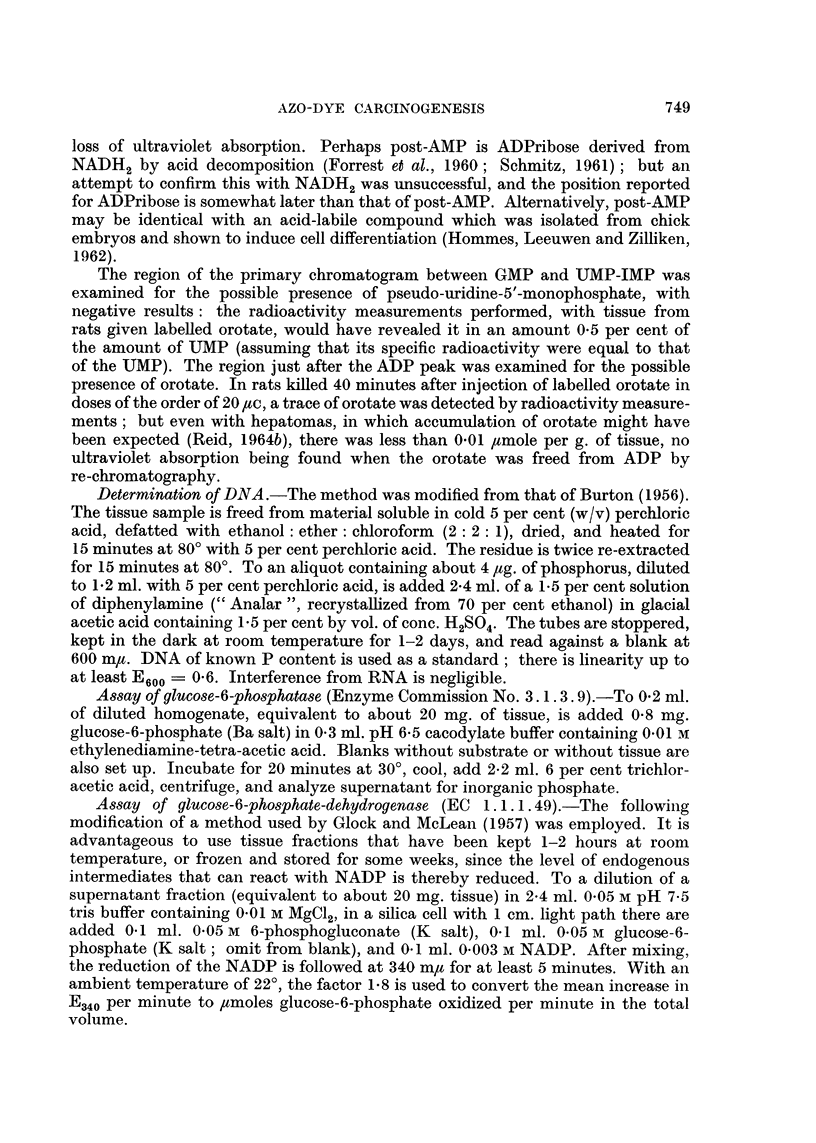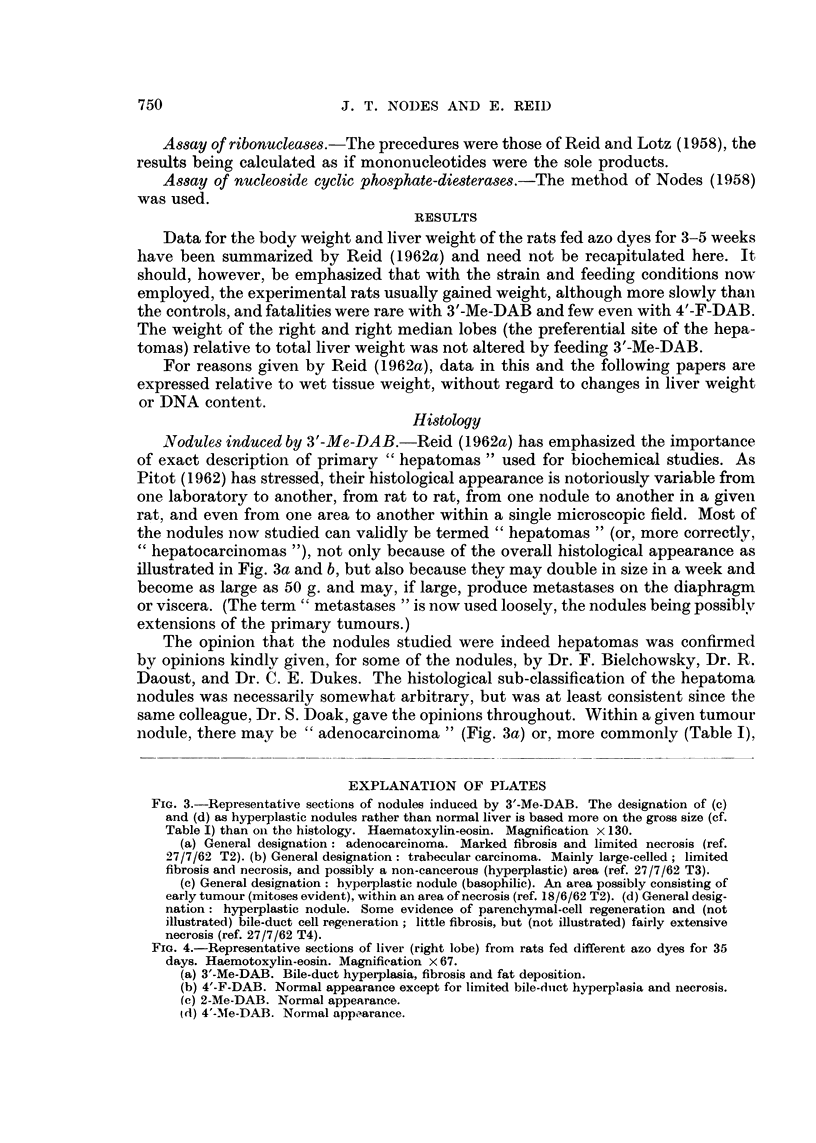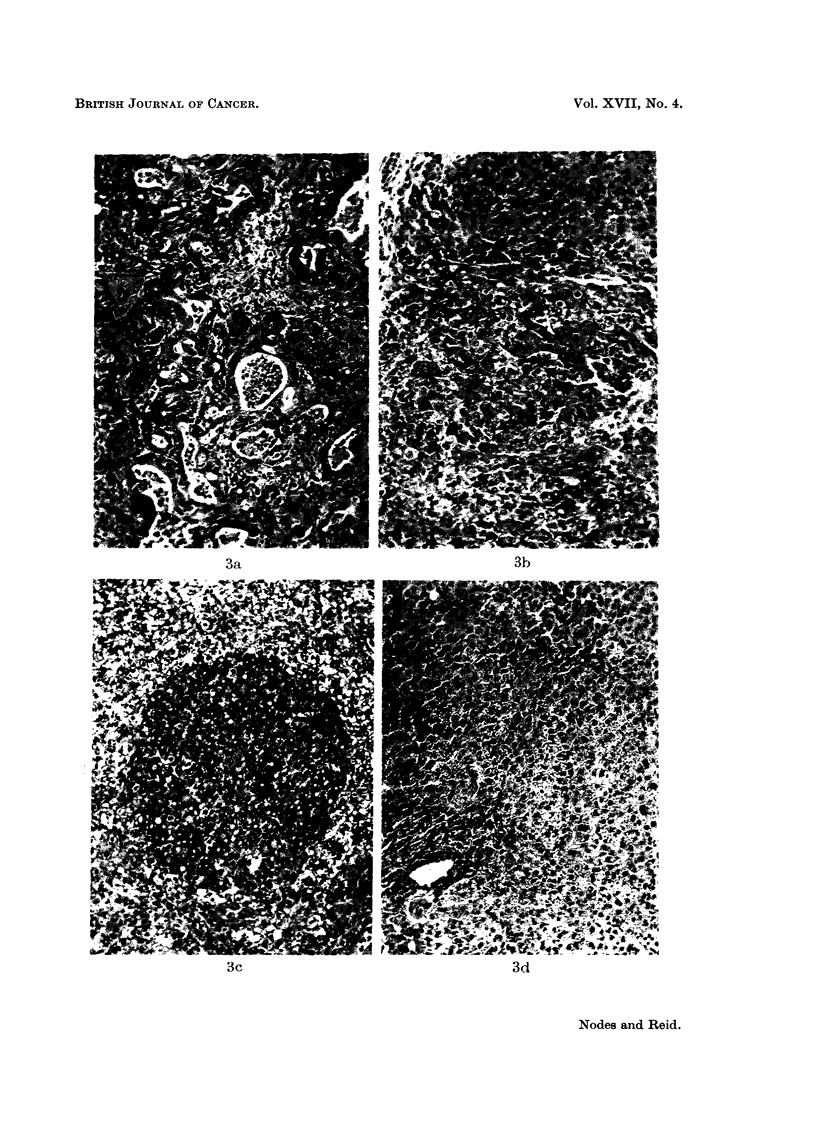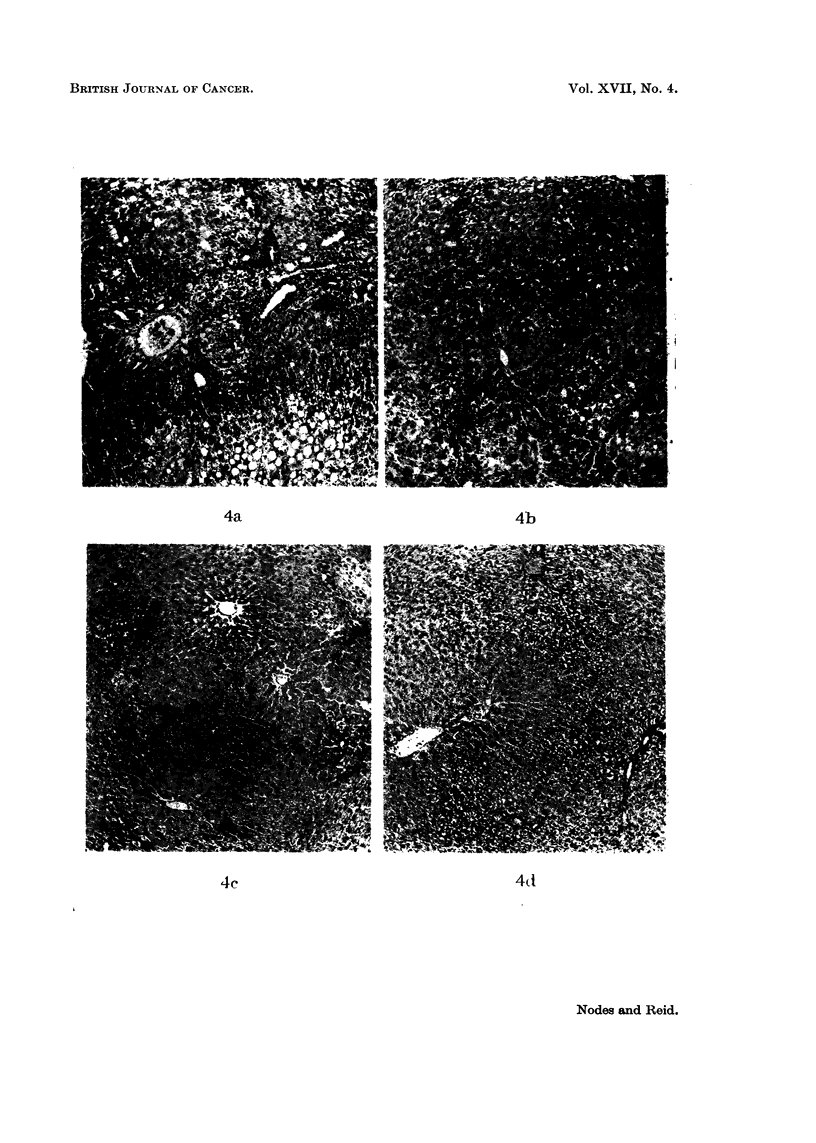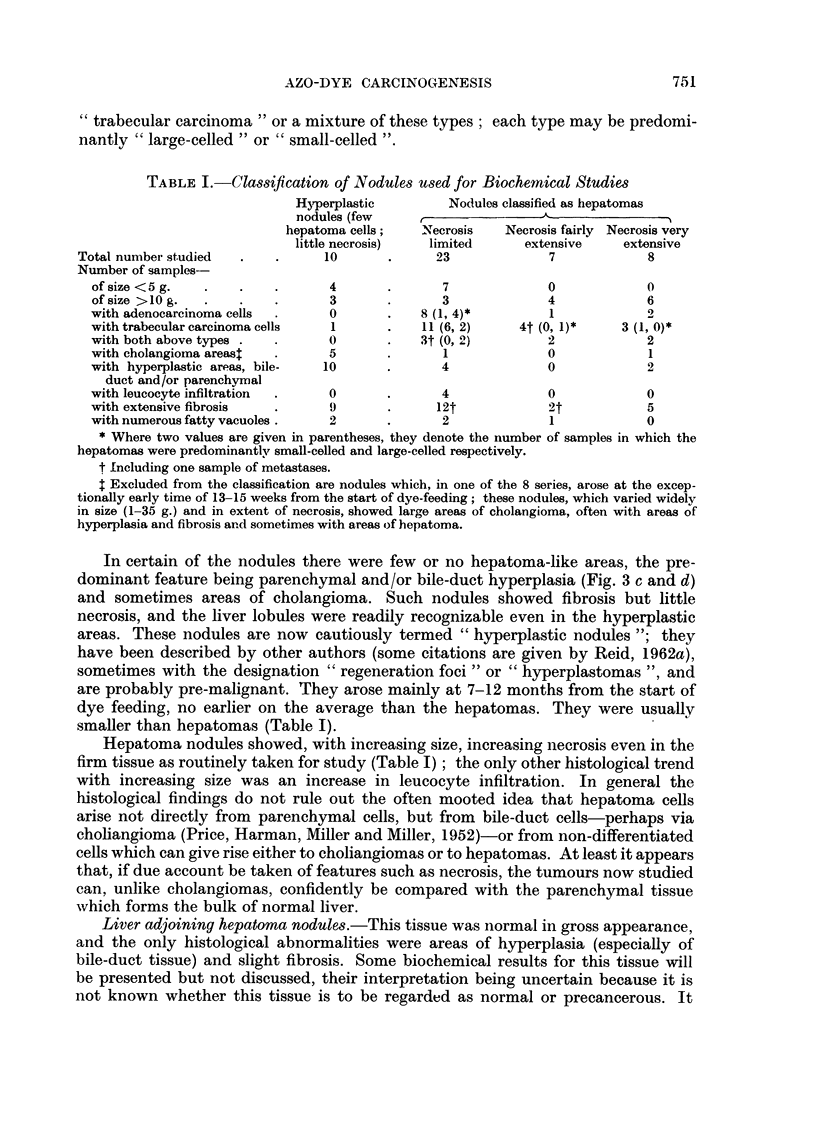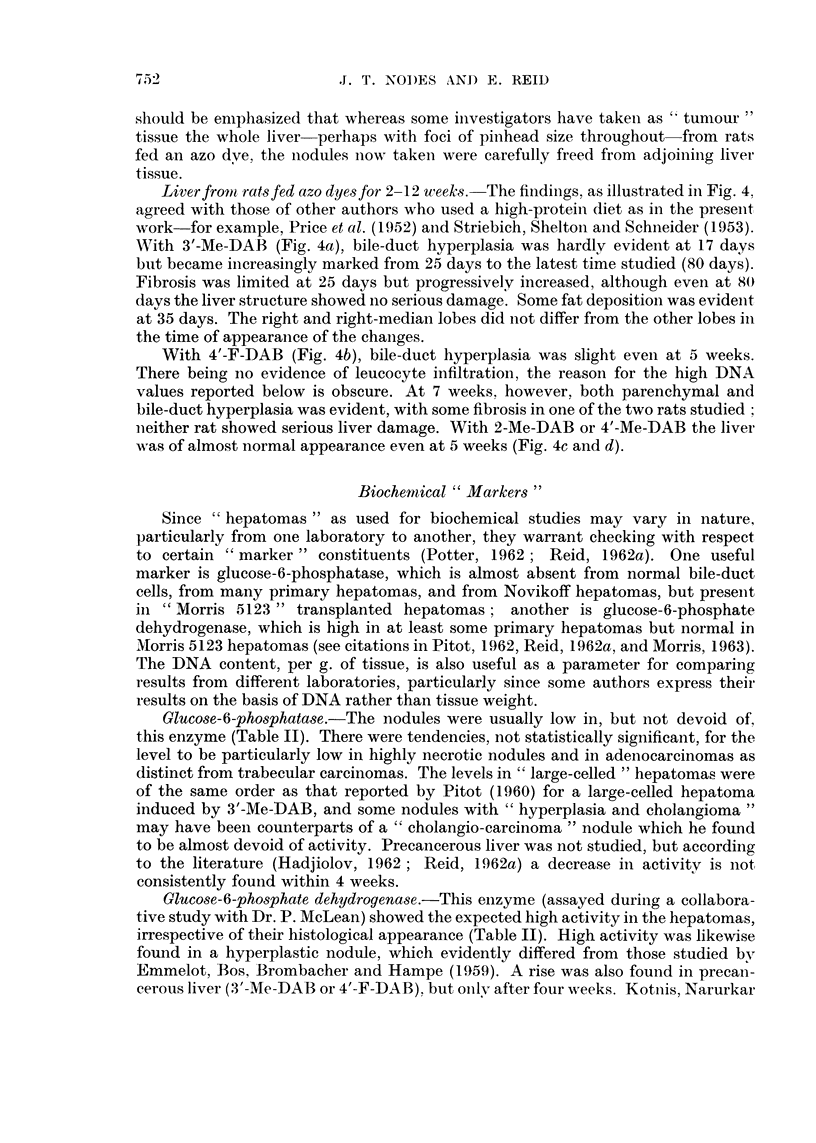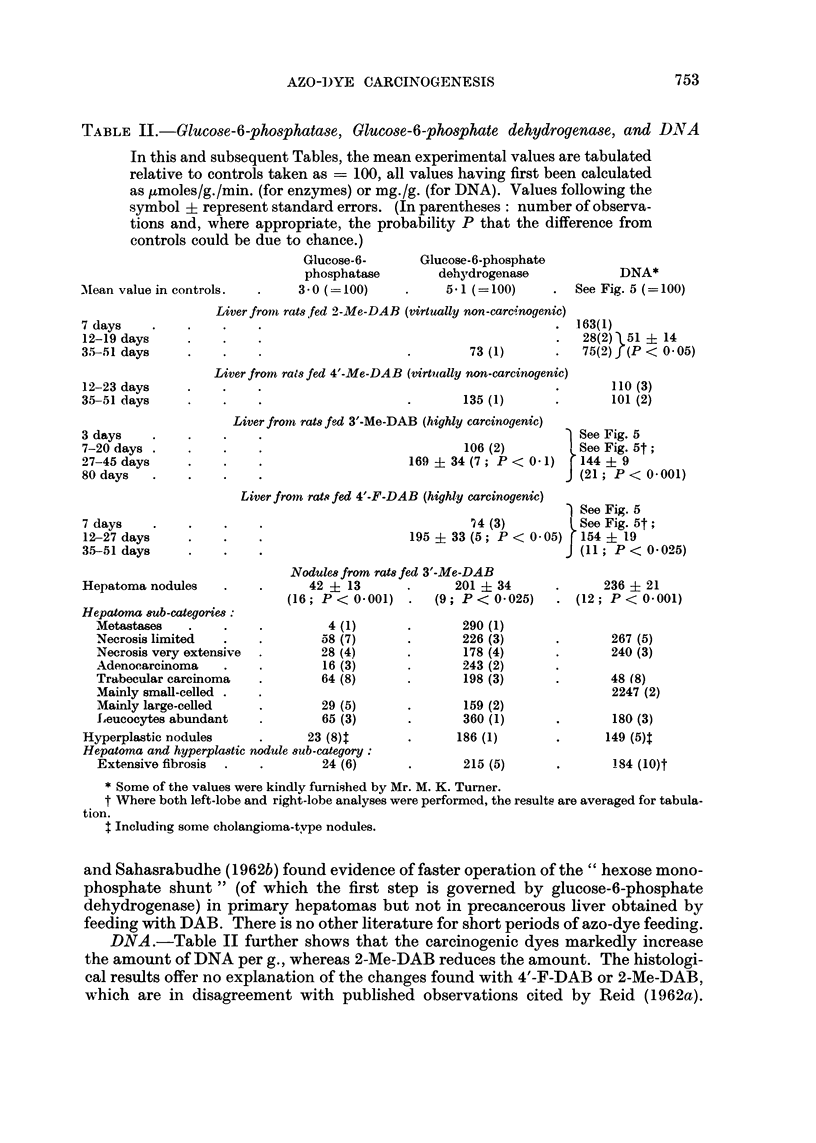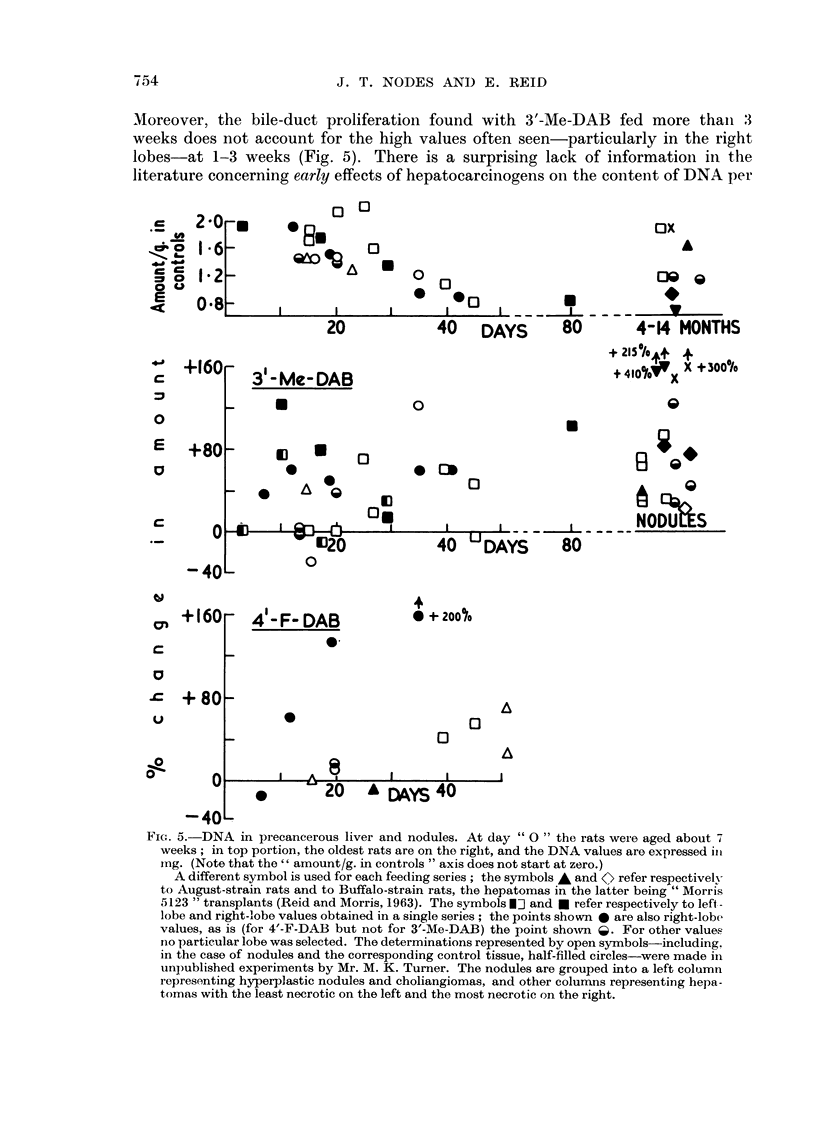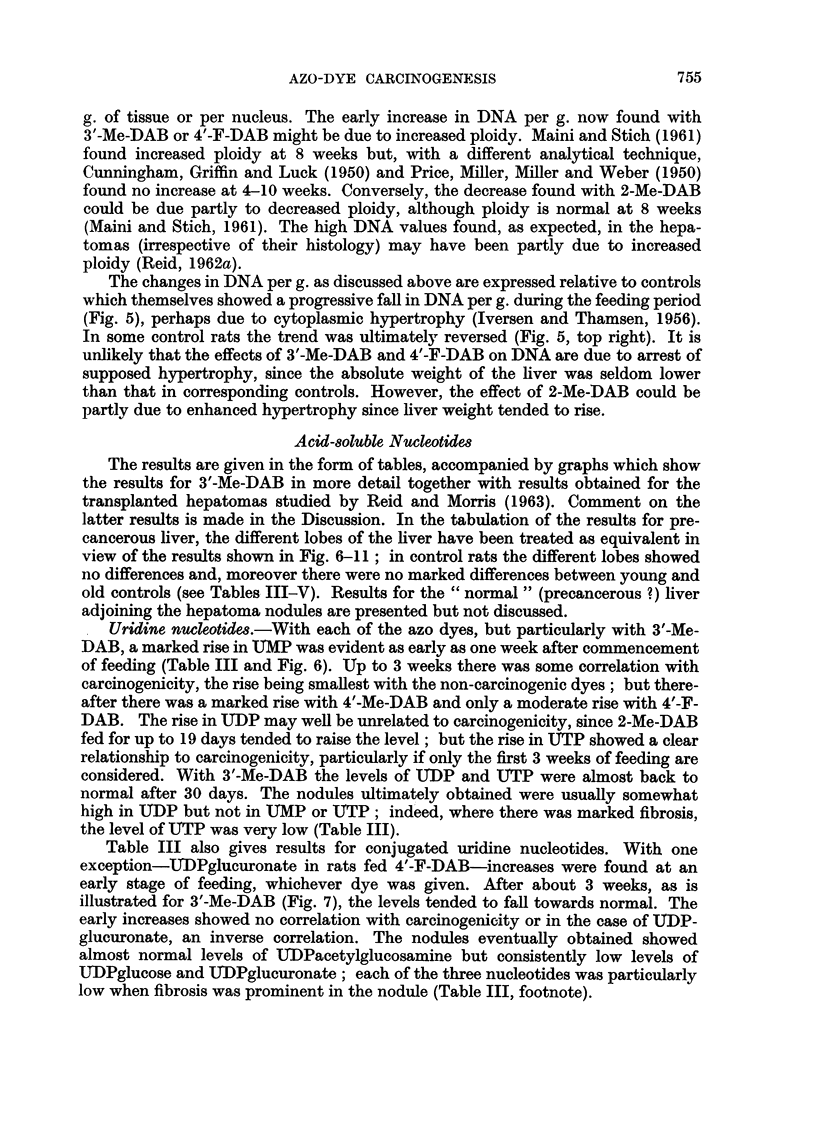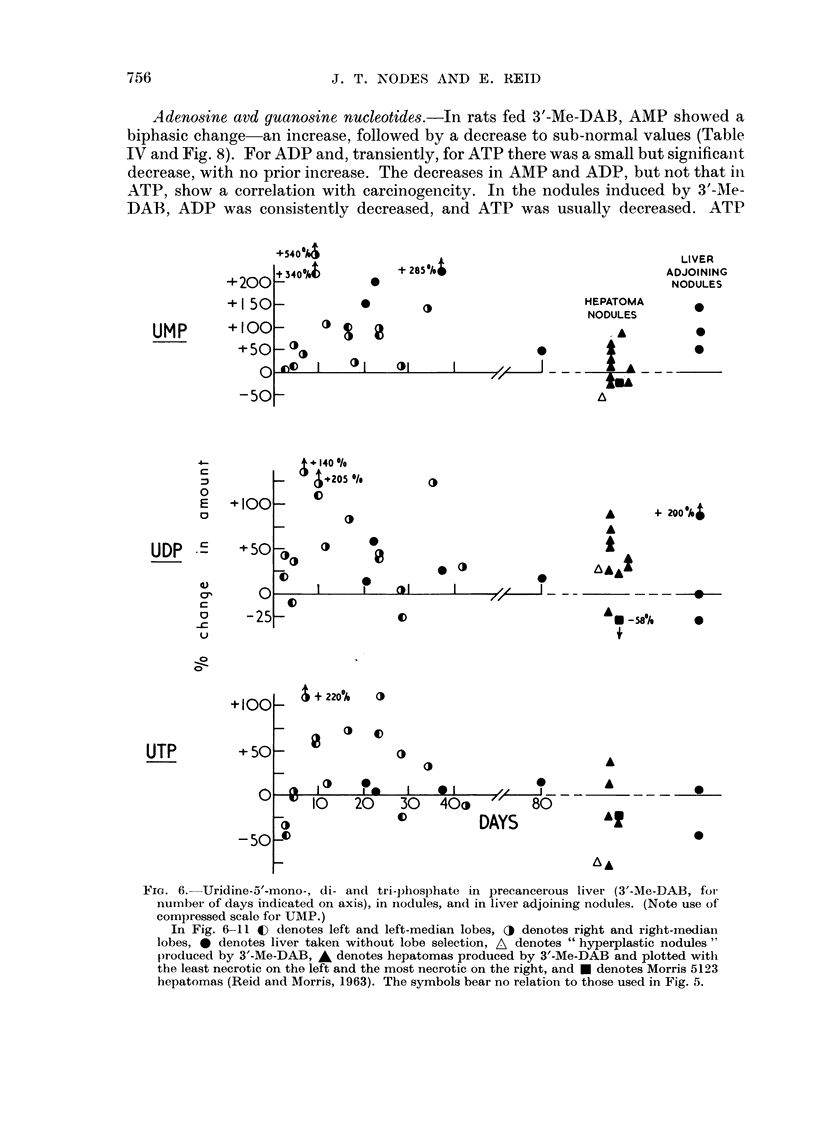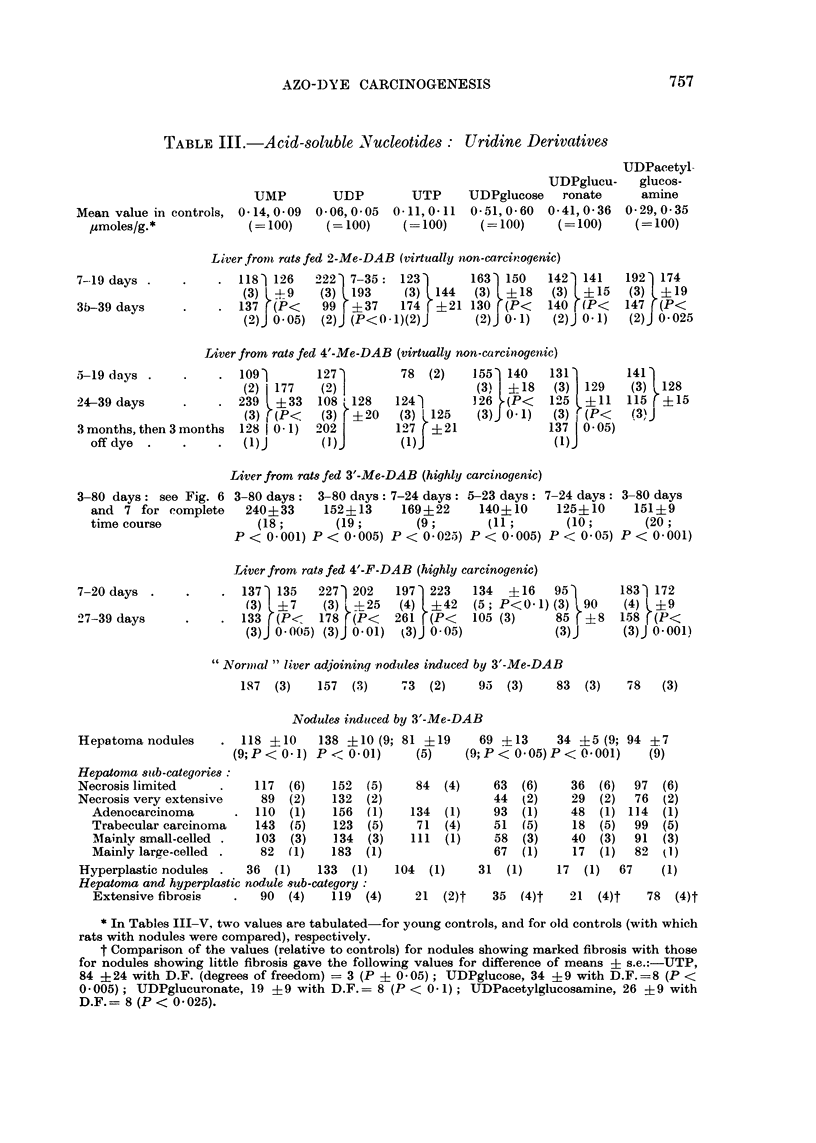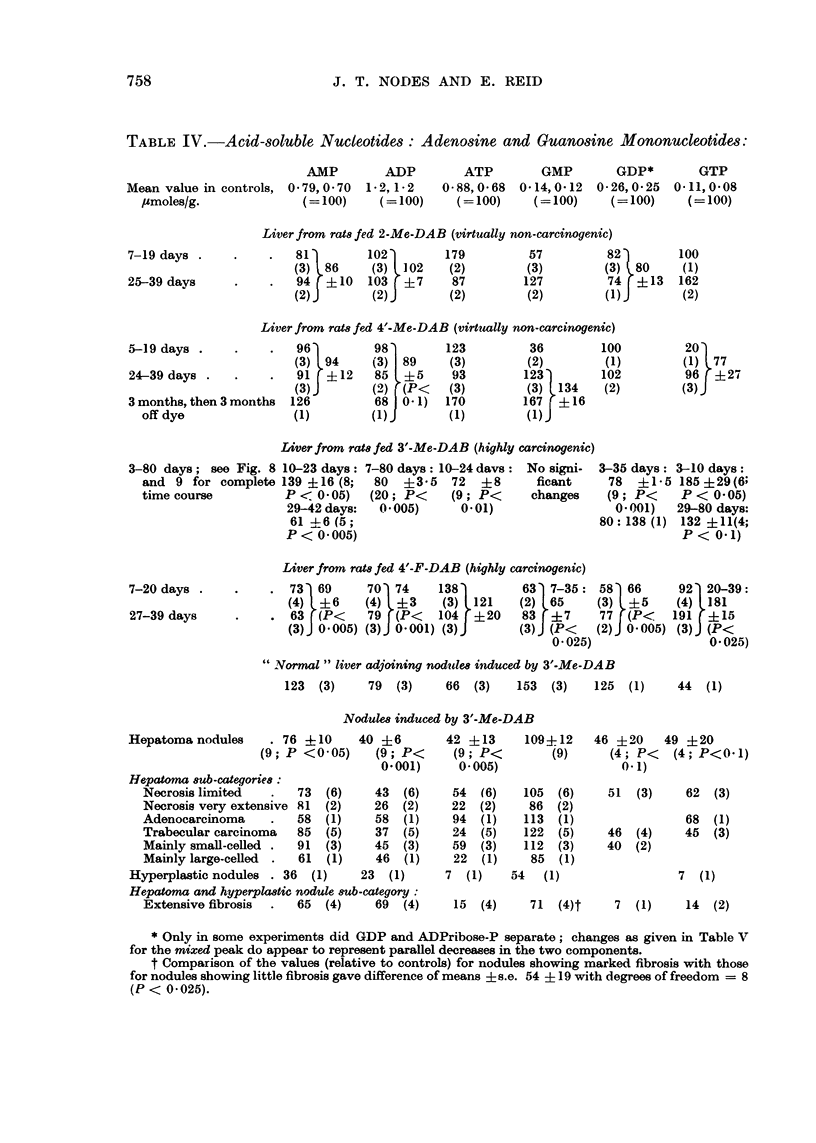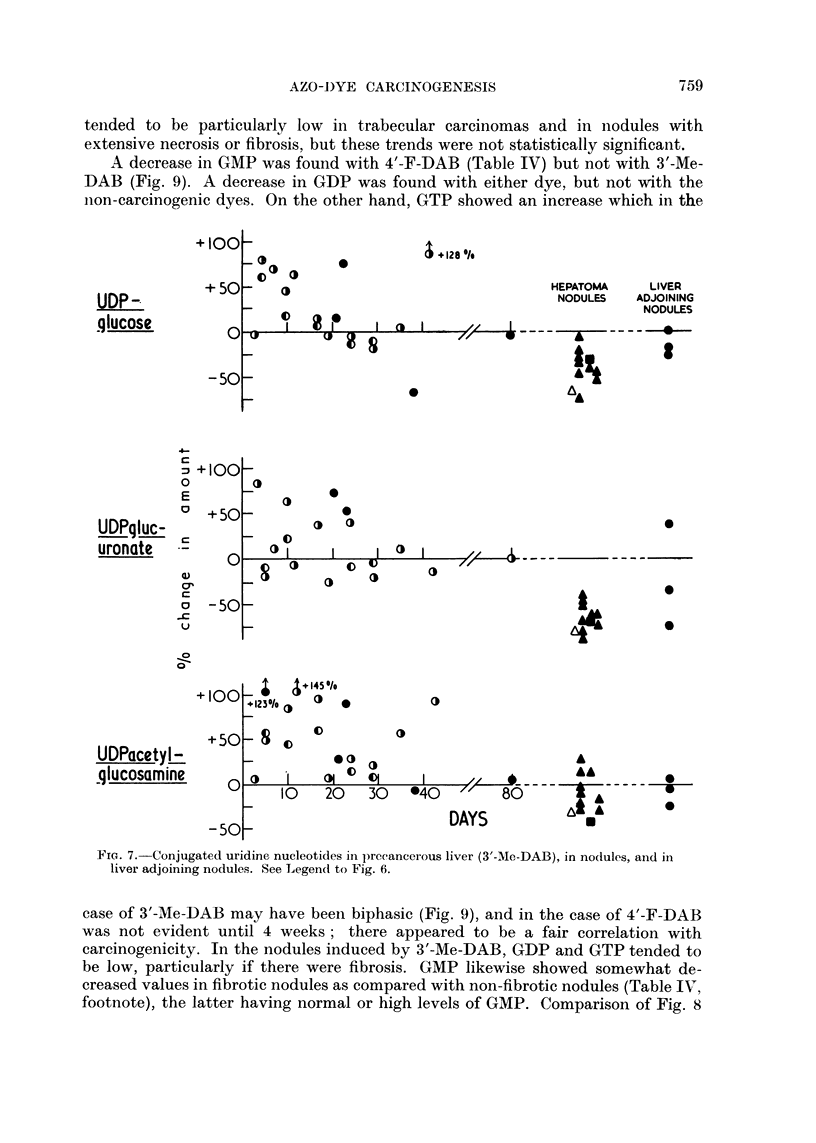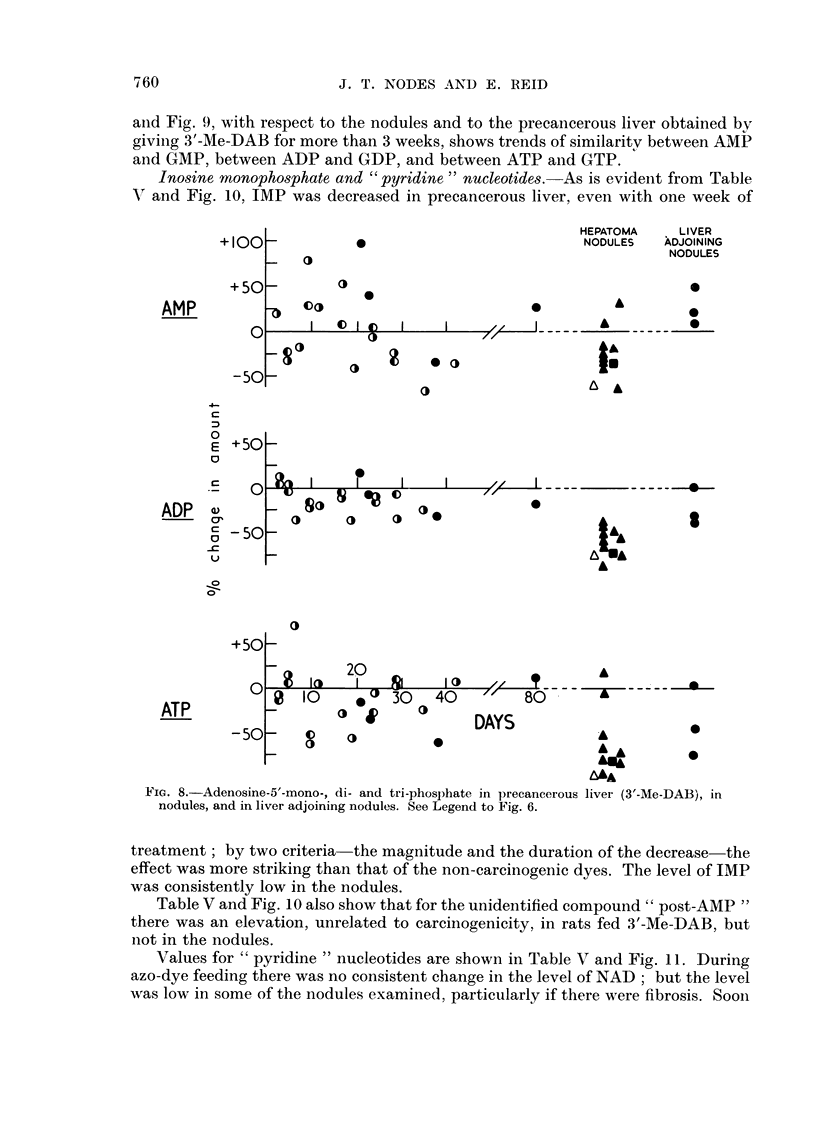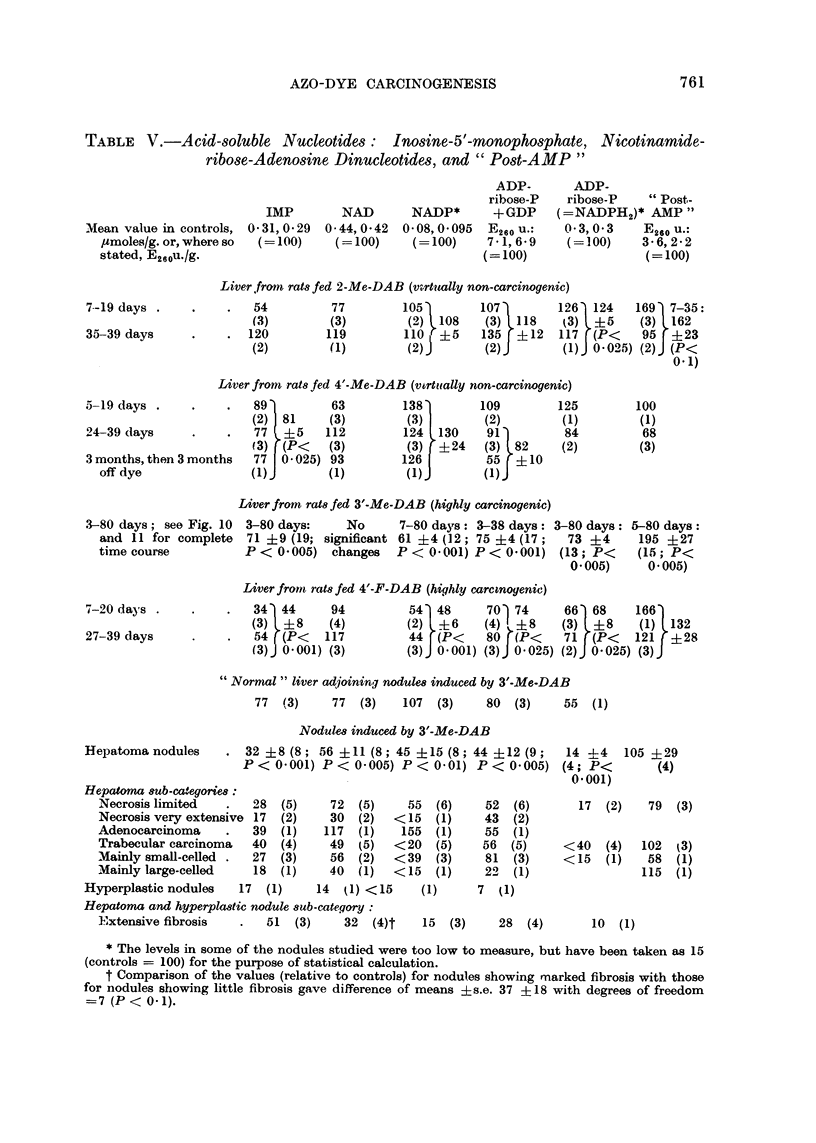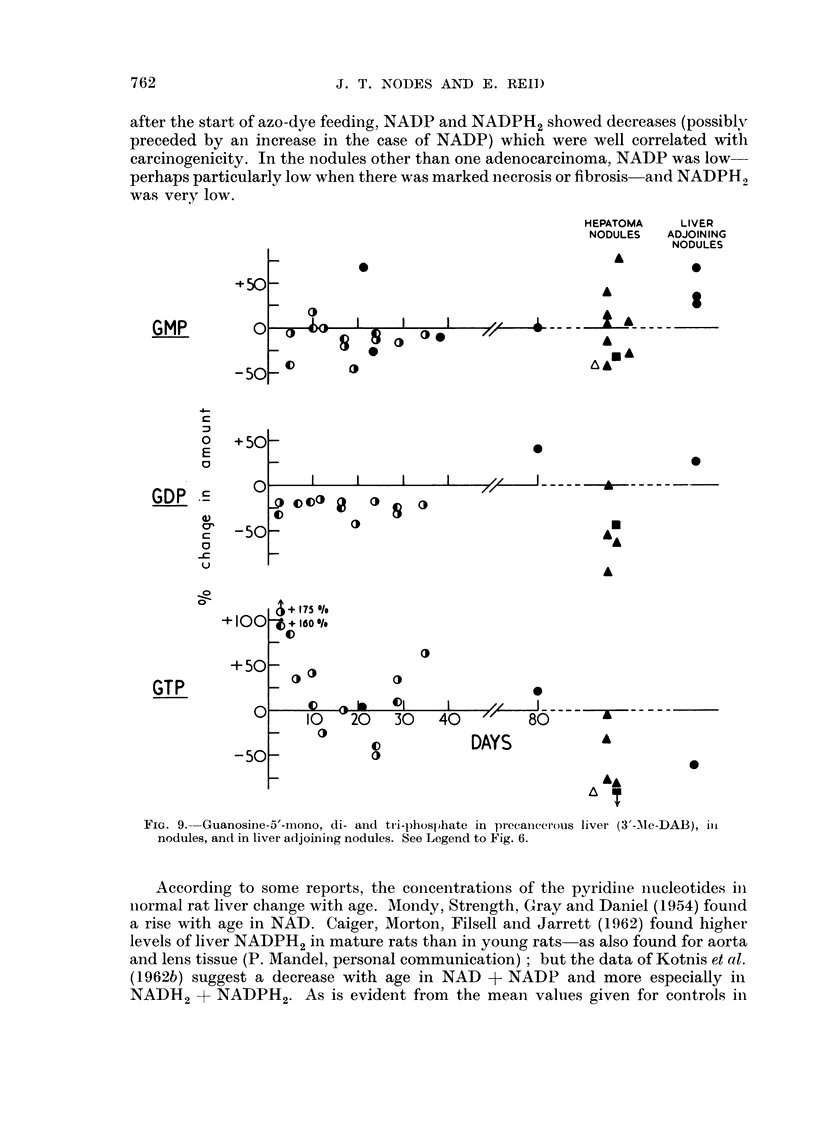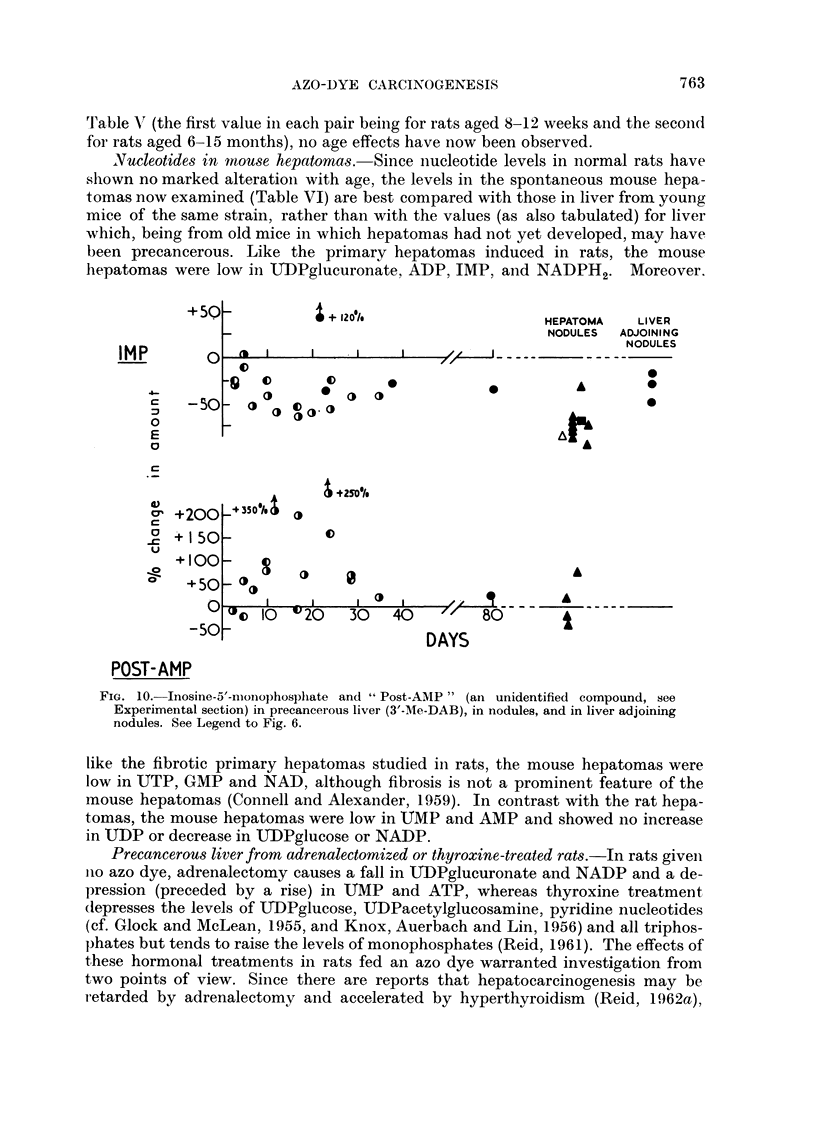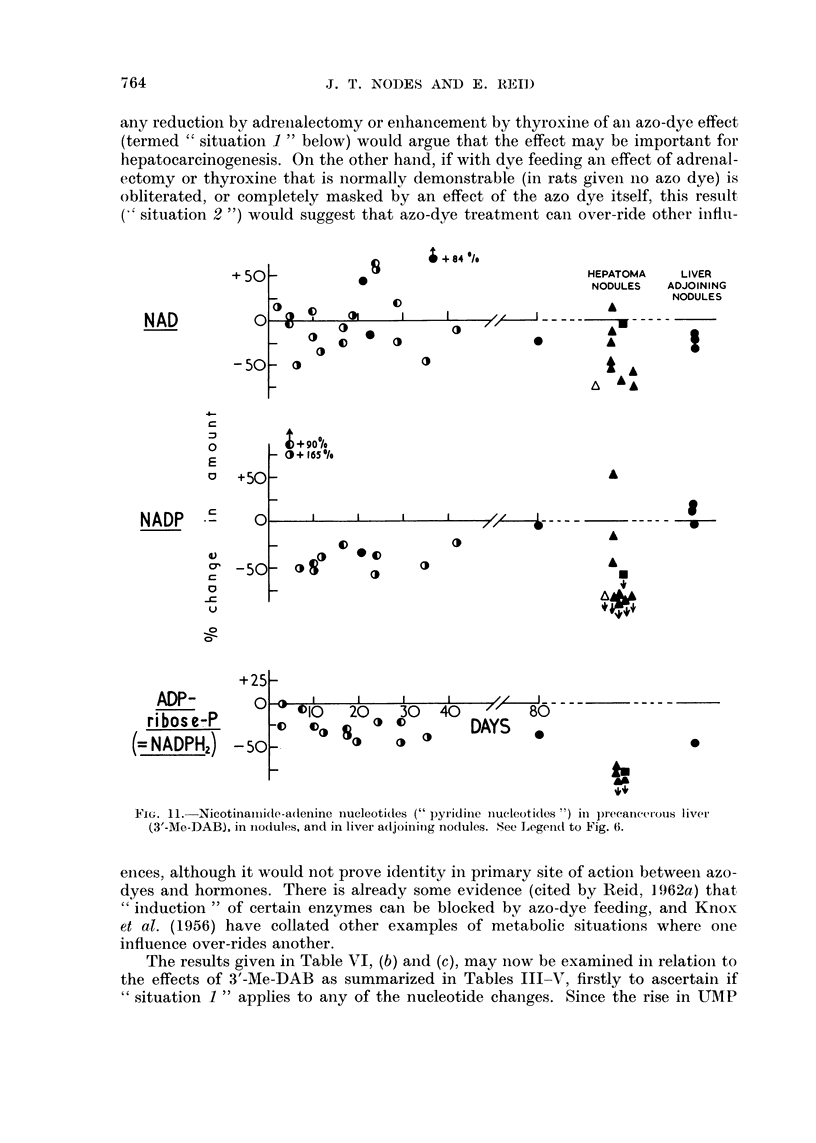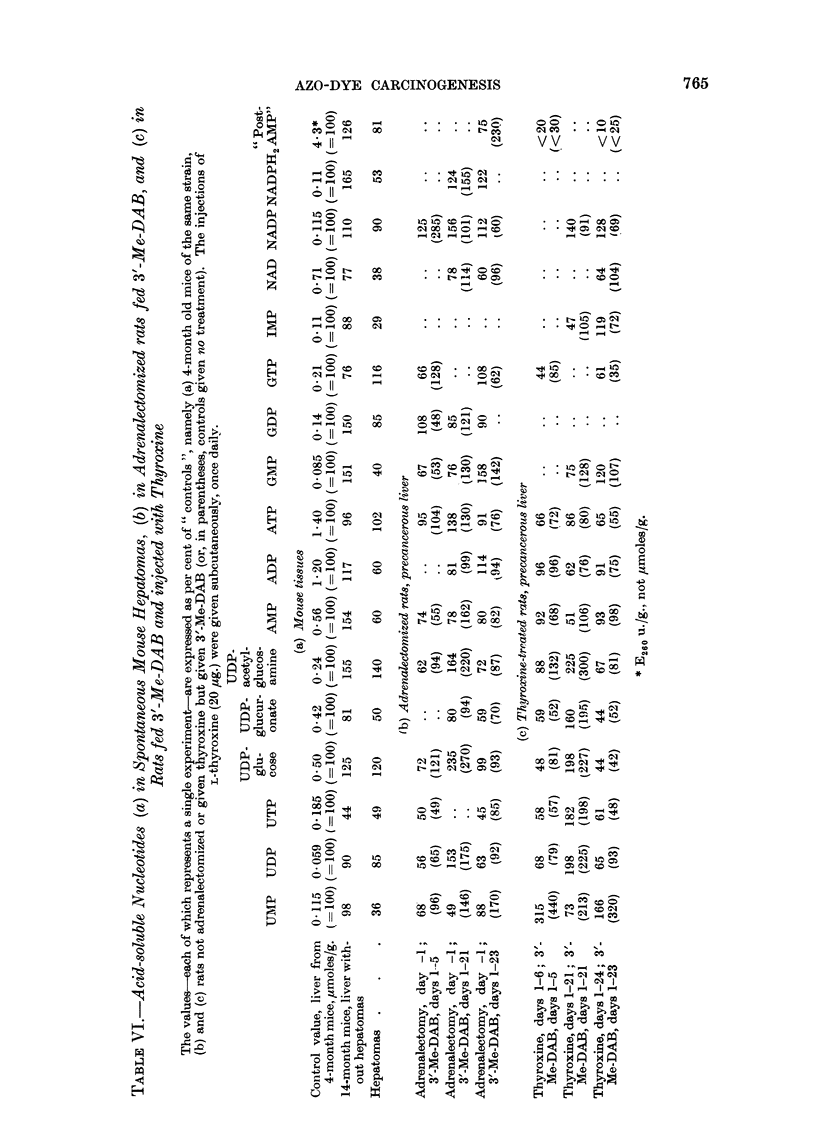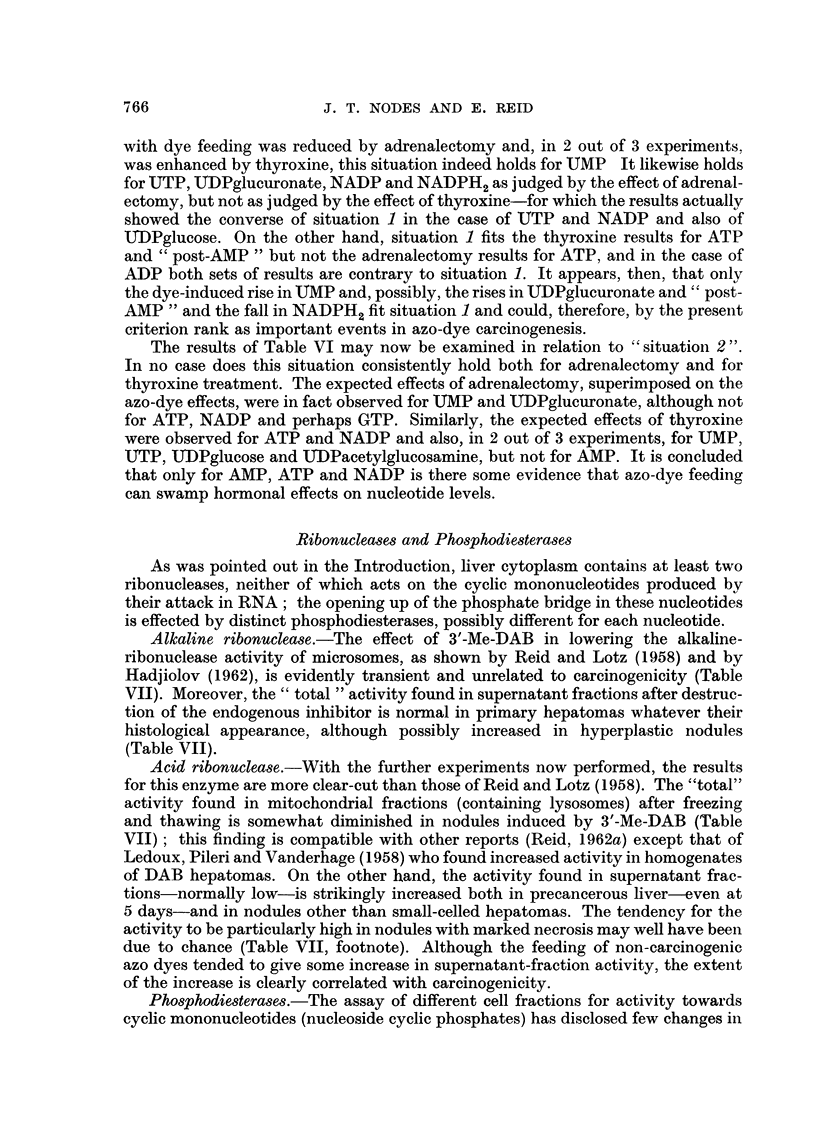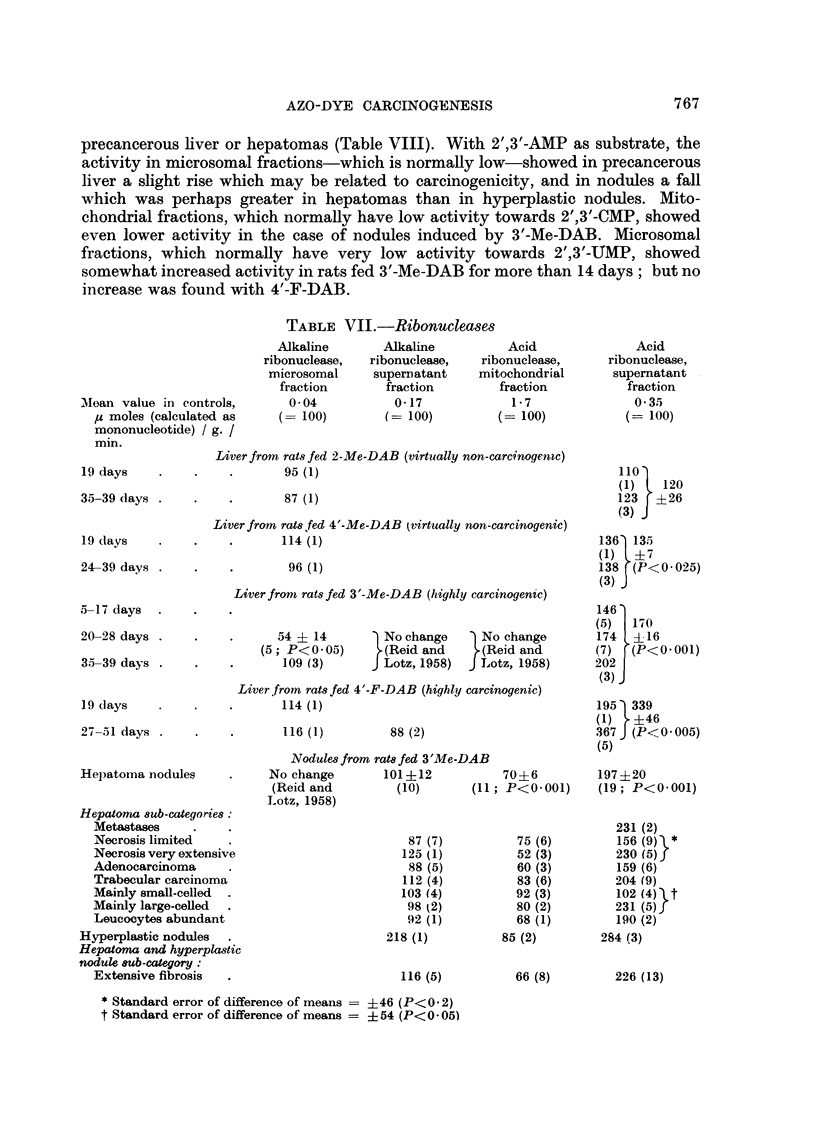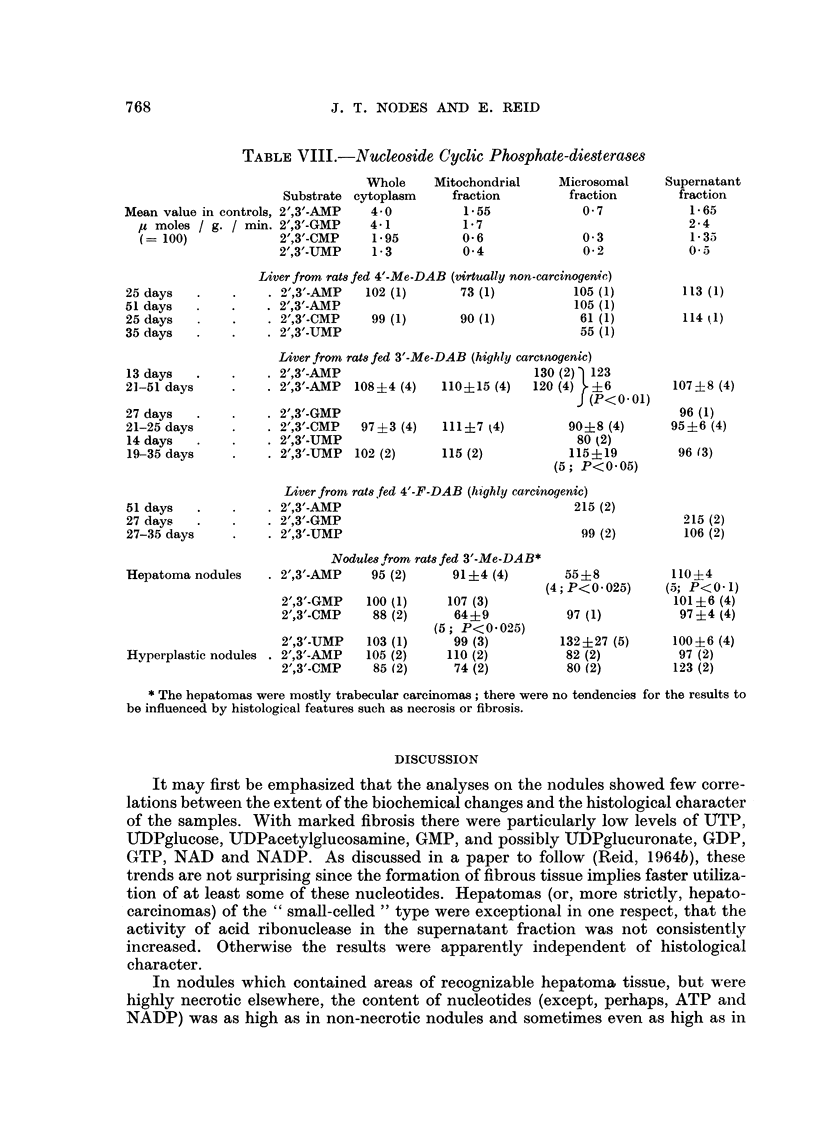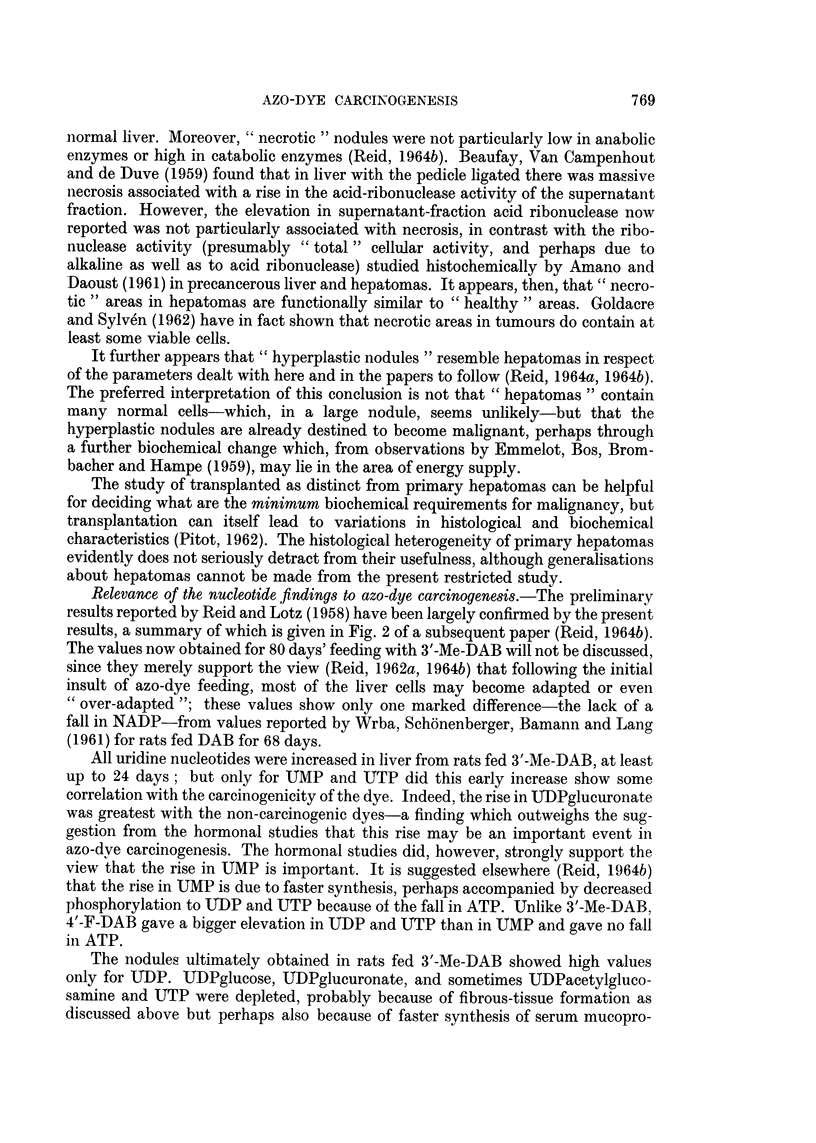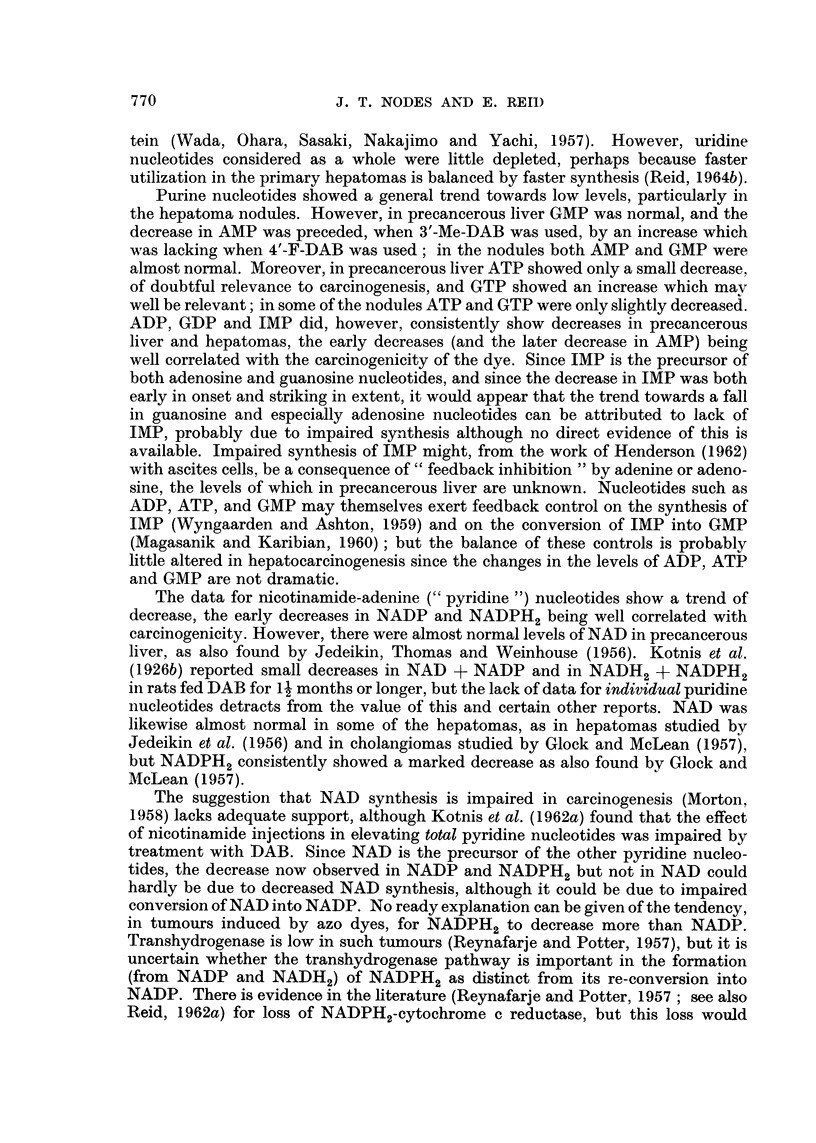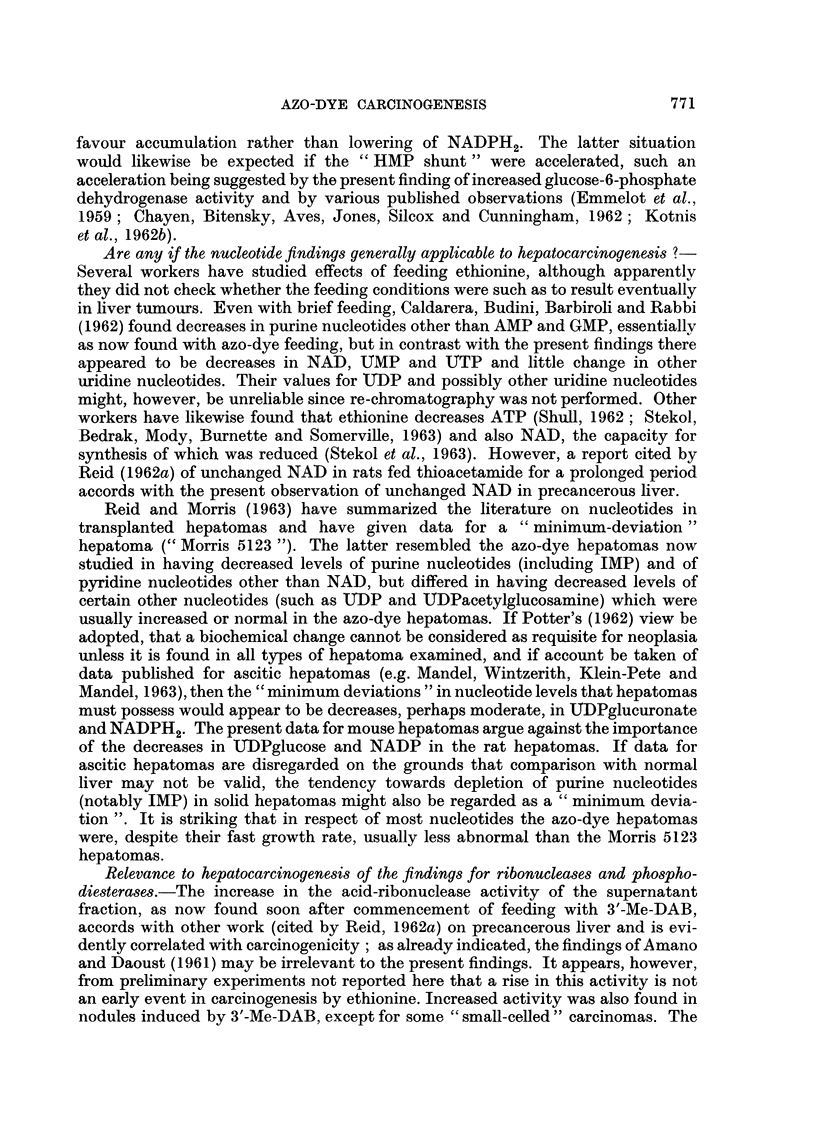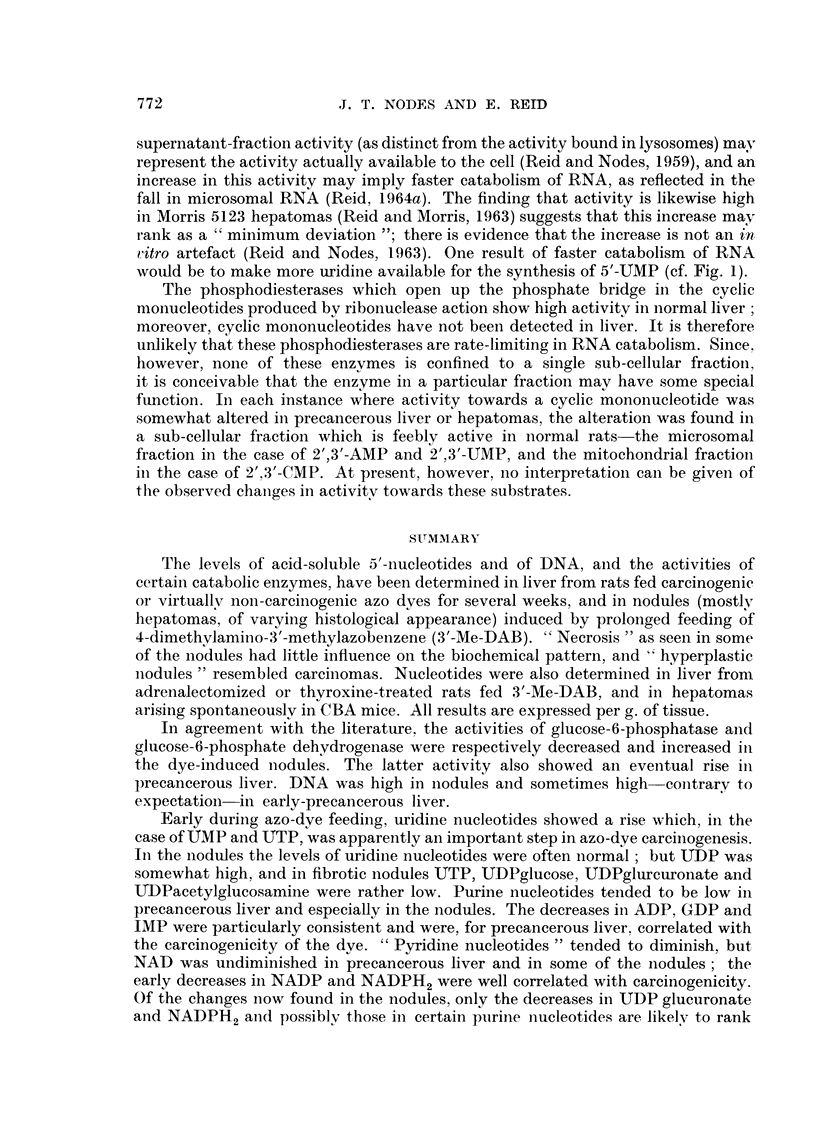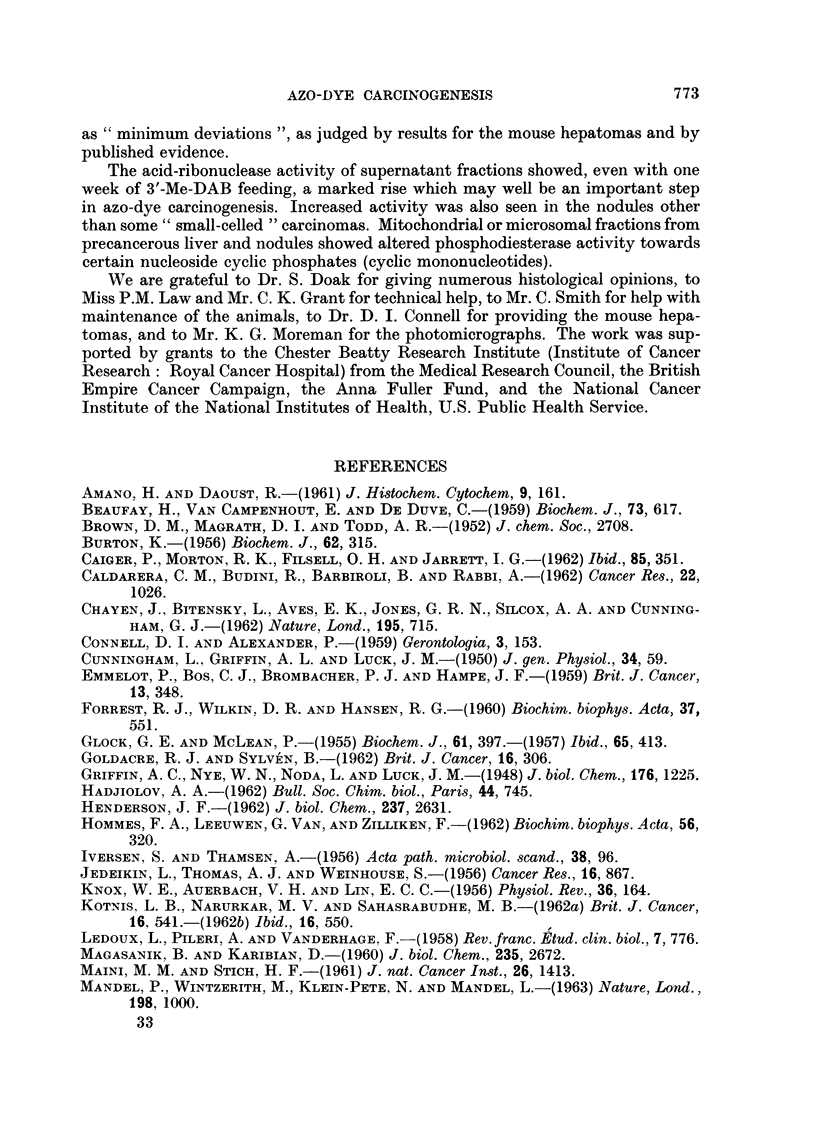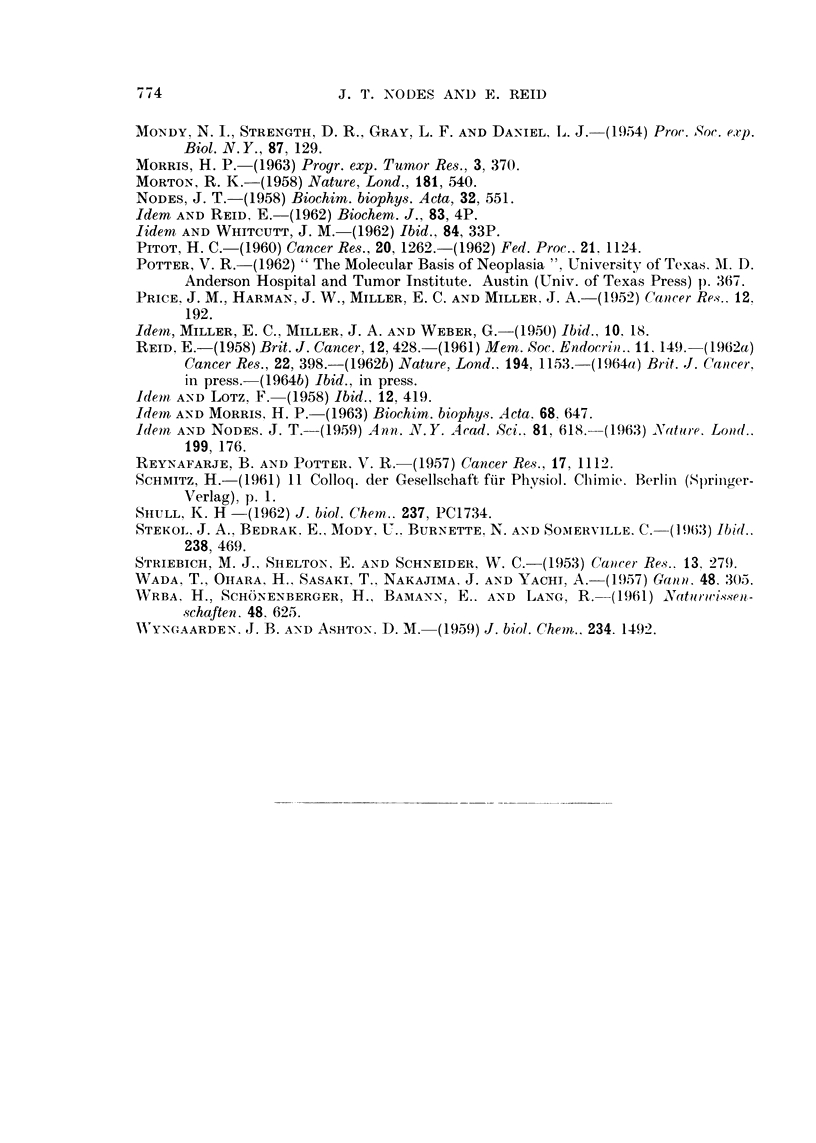# Azo-Dye Carcinogenesis: Ribonucleotides and Ribonucleases

**DOI:** 10.1038/bjc.1963.96

**Published:** 1963-12

**Authors:** J. T. Nodes, E. Reid

## Abstract

**Images:**


### AZO-DYE CARCINOGENESIS: RIBONUCLEOTIDES AND RIBONUCLEASES

J. T. NODES* AND E. REID

*From the Chester Beatty Research Institute of Cancer Research: Royal Cancer Hospital, London, S.W.3*

Received for publication August 1, 1963

IN " precancerous liver " and hepatomas obtained from rats fed a hepatocarcinogen, there are so many biochemical abnormalities that it is difficult to decide which of them reflect fundamental steps in neoplasia (Reid, 1962*a*), although changes not crucial to neoplasia may nevertheless give guidance to pharmacologists (Potter, 1962). For the present purpose of elucidating the mechanism of hepatocarcinogenesis, certain questions can usefully be posed concerning each change observed in hepatoma nodules :—(1) is the change, as observed in the tissue mass examined, truly attributable to living hepatoma cells as distinct from dying cells or non-hepatoma cells in the sample ? (2) is the change, as found in primary hepatomas induced by a particular agent, an irreducible property of all hepatomas? (3) is the change an early event, observable in the " precancerous " liver obtained by feeding the agent for a few weeks only, or a late event associated with the actual appearance of cancer cells ? With precancerous liver further questions arise :—(4) are there changes which are lacking, or converse in direction, in the hepatoma ultimately obtained ? (5) is each observed change specific both in being attributable to liver cells (rather than to bile-duct cells, for example), and in being produced by any hepatocarcinogen and only by hepatocarcinogens?

On the assumption that the nodules now studied, induced by azo-dye feeding, are derived from liver cells and are therefore truly comparable with normal liver, these questions are now approached on the following lines :—(1) biochemical findings have been correlated with histological appearance ; (2) the literature has been surveyed, and a " minimum-deviation hepatoma " (Potter, 1962) given preliminary study (Reid and Morris, 1963) ; (3) and (4) liver has been examined at different stages of azo-dye feeding ; (5) two carcinogenic azo-dyes (differing in their histological effects), and two non-carcinogenic analogues, have been compared, and agents other than azo-dyes are now under test.

Since certain species of ribonucleic acid (RNA) play an important role in the economy of the cell and especially in the transmission of " information ", and since a previous study had given evidence of disturbed RNA metabolism in carcinogenesis by 3′-Me-DAB (Reid and Lotz, 1958 ; Reid, 1958), a closer study has now been made in a metabolic area comprising RNA itself, its breakdown products such as 3′-UMP and uracil, and 5′-UMP and other 5′-ribonucleotides which are important for the biosynthesis not only of RNA but also of other compounds such as glycogen (Fig. 1). If hepatocarcinogens specifically cause changes within this area, these changes might of course be indirect effects ; but they would nevertheless serve as valuable pointers to the underlying primary changes.

* Present address : Dept. of Applied Biology, Brunel College, Acton, London W.3.

In a following paper (Reid, 1964*a*), sub-cellular fractions will be considered with especial reference to their content of RNA and also of protein. The third paper (Reid, 1964*b*) will concern the enzymes which mediate the formation and breakdown of uridine nucleotides. The present paper deals with the histology of the tissue samples examined, with acid-soluble nucleotides (5′-ribonucleotides) as measured in whole tissue, and with the ribonucleases and phosphodiesterases which effect the breakdown of RNA to mono-nucleotides (steps 7 and 8, Fig. 1). A brief report of the changes in acid-soluble nucleotides was given by Nodes and Reid (1962). Of the ribonucleases, acid ribonuclease warranted particular attention. It was known that acid ribonuclease produces nucleoside 2′, 3′-cyclic

FIG. 1.—Field of study. The abbreviations for adenosine (A), guanosine (G), uridine (U) and other nucleotides follow standard biochemical practice, P denotingphosphate (M = mono, D = di, T = tri). The final steps leading to RNA synthesis are shown thus : - - - - - - → ; the participation of cytidine nucleotides is not shown. The reaction steps studied in this paper and in the final paper of the series (Reid, 1964*b*) are denoted by italicized numbers.

phosphates (cyclic nucleotides) but does not attack the latter (Reid and Nodes, 1959 ; Nodes, Reid and Whitcutt, 1962) ; the breakdown of the cyclic nucleotides is effected by phosphodiesterases, which may well be different for each nucleotide.

### EXPERIMENTAL

*Materials and abbreviations.*—For the induction of hepatomas, and for much of the work on precancerous liver, use was made of 3′-methyl-4-dimethylaminoazobenzene (more correctly termed 4-dimethylamino-3′-methylazobenzene ; abbreviated 3′-Me-DAB), as in previous studies from this laboratory (Reid and Lotz, 1958). If a biochemical effect was evident in precancerous liver with 3′-Me-DAB, other azo dyes were tried (see Reid, 1962*a*, for references to their carcinogenicity): —4′-fluoro-4-dimethylaminoazobenzene (4′-F-DAB), 2-methyl-4-dimethylaminoazobenzene (2-Me-DAB), and 4′-methyl-4-dimethylaminoazobenzene (4′-Me-DAB). That 4′-Me-DAB was virtually non-carcinogenic was confirmed by feeding the dye for 137 days to 4 rats ; on autopsy at 15 months the livers were of normal appearance. Mr. J. L. Everett (of this Institute) kindly provided the 4′-F-DAB. Other azo dyes were purchased from L. Light and Co., Colnbrook, Bucks.

Chemicals were of " Analar " or equivalent grade where available. 6-Phosphogluconate was the Ba salt as supplied by Boehringer GmbH (Mannheim, W. Germany), and was converted to the K salt before use. Nucleotides as used in this and the following papers (Reid, 1964*a*, *b*) were usually obtained from the Sigma Chemical Co. (St. Louis, Missouri), but NADP (TPN according to the old nomenclature) was supplied by Boehringer. For nucleotides, use is made of abbreviations as accepted by biochemical journals without definition ; NAD is the dinucleotide termed DPN in the old nomenclature. The nucleotides are the 5′-isomers unless otherwise stated ; for example, UMP denotes uridine-5′-monophosphate, whereas 2′-(3′-)UMP denotes a mixture of the 2′- and 3′-isomers, and 2′,3′-UMP denotes the cyclic nucleotide with phosphate bridging the 2′ and 3′ positions. The substance " post-AMP " is described in the paragraph on chromatographic procedures.

The RNA used for assays of ribonuclease activity was obtained from commercial RNA (yeast) by the phenol procedure, with final dialysis. Cyclic nucleotides were purchased from Schwarz and Co. (Mount Vernon, N.Y.), or were prepared by the method of Brown, Magrath and Todd (1952).

*Animals, injections, and tissue sampling.*—Except for the experiment with CBA male mice, all tissue samples were derived from male rats which were usually of the Institute albino strain. From the age of 7 weeks (body weight about 200 g.) the rats were fed a 20 per cent protein diet containing the azo-dye, as in the experiments of Griffin, Nye, Noda and Luck (1948) but with 0·075 instead of 0·06 per cent of azo dye. The diet was given in weighed amounts, the controls being restricted to the food intake of the experimental rats. Adrenalectomized rats were given saline to drink. On the day before autopsy, the rats were put into individual cages and given a 20 per cent protein diet without azo dye, in restricted amount such that the rats were fasting at the time of autopsy. In rats kept for the study of tumours, the feeding of 3′-Me-DAB was stopped after 90–110 days and stock diet was given *ad lib.* until tumours arose (usually between 7 and 12 months from the start of the dye feeding) ; the latent period was not detectably lengthened by thus discontinuing dye-feeding, but tended to be longer in one experiment where dye feeding was stopped after 58 days. A few of the nodules induced by 3′-Me-DAB were from rats of the August strain, in which the latent period was longer than for the albinos ; no strain difference in the biochemical results was evident.

Tissue samples, which were always taken late in the morning, were plunged into liquid nitrogen if required for nucleotide analyses, or were homogenized in 0·25 M sucrose medium and centrifuged by conventional procedures if subcellular fractions were required (Reid and Lotz, 1958). Where nodules contained a soft centre, this was rejected. From each specimen used for biochemical study, two samples were taken for histological examination and good agreement in appearance was usually found.

### *Estimations*

*Chromatography of nucleotides.*—The procedure for extracting tissue samples in the frozen state and for analysis (by gradient elution on Dowex-1 formate resin as shown in Fig. 2, with re-chromatography of mixed peaks), was as in the experiments of Reid and Lotz (1958). Changes of solvent were made automatically by pre-setting a changing device designed and supplied by the Central Ignition Co., London, N.1. Values for UMP, IMP, UDP, UDPglucuronate, ATP, UTP and GTP are based on re-chromatography, those calculable from the primary chromatogram being seldom reliable. In the re-chromatography there was good recovery of ultraviolet-absorbing material except for the UTP-GTP peak ; the low recovery (40–80 per cent) for the latter could not, however, account for observed differences between experimental and control rats in the levels of UTP and of GTP.

With the batch of resin employed (batch 3807 ; Dow Chemical Co., Midland, Mich.), the NAD peak was consistently contaminated to a small extent with CMP ; some authors have obtained separation—CMP running before NAD according to some reports, and after according to others—but even with 0·04 N formic

FIG. 2.—Characteristic nucleotide pattern in primary chromatogram of extract of normal liver (10 g.). Dowex-1 column 15 × 1 cm.; 500 ml. mixing flask ; 5 ml. collections. F.A. denotes formic acid. Re-chromatography was routinely performed on 3 peaks :—UMP-IMP, UDP-ATP, and UTP-GTP.

acid as the initial solvent, separation could not be achieved. NADP consistently ran ahead of GMP. The " ADX " peak as measured by Reid and Lotz (1958) usually split into two peaks in the present analyses : GDP followed by a peak now known to consist of ADPribose-P. The latter is derived, in stoichiometric amount (as has now been confirmed), from $NADPH_2$ in an acid medium as used for the tissue extraction (Forrest, Wilkin and Hansen, 1960 ; Schmitz, 1961).

Chromatograms from normal liver which had not been stored frozen for a long period usually showed a major and a minor peak of ultraviolet absorption (260 m$\mu$) just after the AMP (Fig. 2). Attempts to identify the major peak, here termed " post-AMP ", have been unsuccessful. It had no radioactivity if isolated from rats given labelled orotate. The crude peak had a variable spectrum although a consistently low $E_{275}/E_{260}$ ratio at pH 4 (about 0·2) ; per unit of $E_{260}$ absorption (1 u corresponds to 1 ml. with $E_{260}$ = unity, 1 cm. light path) there was obtained, on digestion, 0·2 $\mu$moles of phosphate, some of which may have come from contaminating AMP. Freeze-drying led, even with prior neutralisation, to complete loss of ultraviolet absorption. Perhaps post-AMP is ADPribose derived from $NADH_2$ by acid decomposition (Forrest *et al.*, 1960; Schmitz, 1961); but an attempt to confirm this with $NADH_2$ was unsuccessful, and the position reported for ADPribose is somewhat later than that of post-AMP. Alternatively, post-AMP may be identical with an acid-labile compound which was isolated from chick embryos and shown to induce cell differentiation (Hommes, Leeuwen and Zilliken, 1962).

The region of the primary chromatogram between GMP and UMP-IMP was examined for the possible presence of pseudo-uridine-5′-monophosphate, with negative results: the radioactivity measurements performed, with tissue from rats given labelled orotate, would have revealed it in an amount 0·5 per cent of the amount of UMP (assuming that its specific radioactivity were equal to that of the UMP). The region just after the ADP peak was examined for the possible presence of orotate. In rats killed 40 minutes after injection of labelled orotate in doses of the order of 20 $\mu$c, a trace of orotate was detected by radioactivity measurements; but even with hepatomas, in which accumulation of orotate might have been expected (Reid, 1964*b*), there was less than 0·01 $\mu$mole per g. of tissue, no ultraviolet absorption being found when the orotate was freed from ADP by re-chromatography.

*Determination of DNA.*—The method was modified from that of Burton (1956). The tissue sample is freed from material soluble in cold 5 per cent (w/v) perchloric acid, defatted with ethanol : ether : chloroform (2 : 2 : 1), dried, and heated for 15 minutes at 80° with 5 per cent perchloric acid. The residue is twice re-extracted for 15 minutes at 80°. To an aliquot containing about 4 $\mu$g. of phosphorus, diluted to 1·2 ml. with 5 per cent perchloric acid, is added 2·4 ml. of a 1·5 per cent solution of diphenylamine ("Analar", recrystallized from 70 per cent ethanol) in glacial acetic acid containing 1·5 per cent by vol. of conc. $H_2SO_4$. The tubes are stoppered, kept in the dark at room temperature for 1–2 days, and read against a blank at 600 m$\mu$. DNA of known P content is used as a standard; there is linearity up to at least $E_{600} = 0{\cdot}6$. Interference from RNA is negligible.

*Assay of glucose-6-phosphatase* (Enzyme Commission No. 3.1.3.9).—To 0·2 ml. of diluted homogenate, equivalent to about 20 mg. of tissue, is added 0·8 mg. glucose-6-phosphate (Ba salt) in 0·3 ml. pH 6·5 cacodylate buffer containing 0·01 M ethylenediamine-tetra-acetic acid. Blanks without substrate or without tissue are also set up. Incubate for 20 minutes at 30°, cool, add 2·2 ml. 6 per cent trichloracetic acid, centrifuge, and analyze supernatant for inorganic phosphate.

*Assay of glucose-6-phosphate-dehydrogenase* (EC 1.1.1.49).—The following modification of a method used by Glock and McLean (1957) was employed. It is advantageous to use tissue fractions that have been kept 1–2 hours at room temperature, or frozen and stored for some weeks, since the level of endogenous intermediates that can react with NADP is thereby reduced. To a dilution of a supernatant fraction (equivalent to about 20 mg. tissue) in 2·4 ml. 0·05 M pH 7·5 tris buffer containing 0·01 M $MgCl_2$, in a silica cell with 1 cm. light path there are added 0·1 ml. 0·05 M 6-phosphogluconate (K salt), 0·1 ml. 0·05 M glucose-6-phosphate (K salt; omit from blank), and 0·1 ml. 0·003 M NADP. After mixing, the reduction of the NADP is followed at 340 m$\mu$ for at least 5 minutes. With an ambient temperature of 22°, the factor 1·8 is used to convert the mean increase in $E_{340}$ per minute to $\mu$moles glucose-6-phosphate oxidized per minute in the total volume.

*Assay of ribonucleases.*—The precedures were those of Reid and Lotz (1958), the results being calculated as if mononucleotides were the sole products.

*Assay of nucleoside cyclic phosphate-diesterases.*—The method of Nodes (1958) was used.

### RESULTS

Data for the body weight and liver weight of the rats fed azo dyes for 3–5 weeks have been summarized by Reid (1962*a*) and need not be recapitulated here. It should, however, be emphasized that with the strain and feeding conditions now employed, the experimental rats usually gained weight, although more slowly than the controls, and fatalities were rare with 3′-Me-DAB and few even with 4′-F-DAB. The weight of the right and right median lobes (the preferential site of the hepatomas) relative to total liver weight was not altered by feeding 3′-Me-DAB.

For reasons given by Reid (1962*a*), data in this and the following papers are expressed relative to wet tissue weight, without regard to changes in liver weight or DNA content.

### *Histology*

*Nodules induced by 3′-Me-DAB.*—Reid (1962*a*) has emphasized the importance of exact description of primary " hepatomas " used for biochemical studies. As Pitot (1962) has stressed, their histological appearance is notoriously variable from one laboratory to another, from rat to rat, from one nodule to another in a given rat, and even from one area to another within a single microscopic field. Most of the nodules now studied can validly be termed " hepatomas " (or, more correctly, " hepatocarcinomas "), not only because of the overall histological appearance as illustrated in Fig. 3*a* and *b*, but also because they may double in size in a week and become as large as 50 g. and may, if large, produce metastases on the diaphragm or viscera. (The term " metastases " is now used loosely, the nodules being possibly extensions of the primary tumours.)

The opinion that the nodules studied were indeed hepatomas was confirmed by opinions kindly given, for some of the nodules, by Dr. F. Bielchowsky, Dr. R. Daoust, and Dr. C. E. Dukes. The histological sub-classification of the hepatoma nodules was necessarily somewhat arbitrary, but was at least consistent since the same colleague, Dr. S. Doak, gave the opinions throughout. Within a given tumour nodule, there may be " adenocarcinoma " (Fig. 3*a*) or, more commonly (Table I),

---

EXPLANATION OF PLATES

FIG. 3.—Representative sections of nodules induced by 3′-Me-DAB. The designation of (c) and (d) as hyperplastic nodules rather than normal liver is based more on the gross size (cf. Table I) than on the histology. Haematoxylin-eosin. Magnification ×130.

(a) General designation : adenocarcinoma. Marked fibrosis and limited necrosis (ref. 27/7/62 T2). (b) General designation : trabecular carcinoma. Mainly large-celled ; limited fibrosis and necrosis, and possibly a non-cancerous (hyperplastic) area (ref. 27/7/62 T3).

(c) General designation : hyperplastic nodule (basophilic). An area possibly consisting of early tumour (mitoses evident), within an area of necrosis (ref. 18/6/62 T2). (d) General designation : hyperplastic nodule. Some evidence of parenchymal-cell regeneration and (not illustrated) bile-duct cell regeneration ; little fibrosis, but (not illustrated) fairly extensive necrosis (ref. 27/7/62 T4).

FIG. 4.—Representative sections of liver (right lobe) from rats fed different azo dyes for 35 days. Haemotoxylin-eosin. Magnification ×67.

(a) 3′-Me-DAB. Bile-duct hyperplasia, fibrosis and fat deposition.
(b) 4′-F-DAB. Normal appearance except for limited bile-duct hyperplasia and necrosis.
(c) 2-Me-DAB. Normal appearance.
(d) 4′-Me-DAB. Normal appearance.

3a

3b

3c

3d

Nodes and Reid.

4a 4b 4c 4d

" trabecular carcinoma " or a mixture of these types ; each type may be predominantly " large-celled " or " small-celled ".

TABLE I.—*Classification of Nodules used for Biochemical Studies*

| | Hyperplastic nodules (few hepatoma cells ; little necrosis) | Nodules classified as hepatomas: Necrosis limited | Necrosis fairly extensive | Necrosis very extensive |
|---|---|---|---|---|
| Total number studied | 10 | 23 | 7 | 8 |
| Number of samples— | | | | |
| of size <5 g. | 4 | 7 | 0 | 0 |
| of size >10 g. | 3 | 3 | 4 | 6 |
| with adenocarcinoma cells | 0 | 8 (1, 4)* | 1 | 2 |
| with trabecular carcinoma cells | 1 | 11 (6, 2) | 4† (0, 1)* | 3 (1, 0)* |
| with both above types | 0 | 3† (0, 2) | 2 | 2 |
| with cholangioma areas‡ | 5 | 1 | 0 | 1 |
| with hyperplastic areas, bile-duct and/or parenchymal | 10 | 4 | 0 | 2 |
| with leucocyte infiltration | 0 | 4 | 0 | 0 |
| with extensive fibrosis | 9 | 12† | 2† | 5 |
| with numerous fatty vacuoles | 2 | 2 | 1 | 0 |

* Where two values are given in parentheses, they denote the number of samples in which the hepatomas were predominantly small-celled and large-celled respectively.

† Including one sample of metastases.

‡ Excluded from the classification are nodules which, in one of the 8 series, arose at the exceptionally early time of 13–15 weeks from the start of dye-feeding ; these nodules, which varied widely in size (1–35 g.) and in extent of necrosis, showed large areas of cholangioma, often with areas of hyperplasia and fibrosis and sometimes with areas of hepatoma.

In certain of the nodules there were few or no hepatoma-like areas, the predominant feature being parenchymal and/or bile-duct hyperplasia (Fig. 3 *c* and *d*) and sometimes areas of cholangioma. Such nodules showed fibrosis but little necrosis, and the liver lobules were readily recognizable even in the hyperplastic areas. These nodules are now cautiously termed " hyperplastic nodules "; they have been described by other authors (some citations are given by Reid, 1962*a*), sometimes with the designation " regeneration foci " or " hyperplastomas ", and are probably pre-malignant. They arose mainly at 7–12 months from the start of dye feeding, no earlier on the average than the hepatomas. They were usually smaller than hepatomas (Table I).

Hepatoma nodules showed, with increasing size, increasing necrosis even in the firm tissue as routinely taken for study (Table I) ; the only other histological trend with increasing size was an increase in leucocyte infiltration. In general the histological findings do not rule out the often mooted idea that hepatoma cells arise not directly from parenchymal cells, but from bile-duct cells—perhaps via choliangioma (Price, Harman, Miller and Miller, 1952)—or from non-differentiated cells which can give rise either to choliangiomas or to hepatomas. At least it appears that, if due account be taken of features such as necrosis, the tumours now studied can, unlike cholangiomas, confidently be compared with the parenchymal tissue which forms the bulk of normal liver.

*Liver adjoining hepatoma nodules.*—This tissue was normal in gross appearance, and the only histological abnormalities were areas of hyperplasia (especially of bile-duct tissue) and slight fibrosis. Some biochemical results for this tissue will be presented but not discussed, their interpretation being uncertain because it is not known whether this tissue is to be regarded as normal or precancerous. It should be emphasized that whereas some investigators have taken as " tumour " tissue the whole liver—perhaps with foci of pinhead size throughout—from rats fed an azo dye, the nodules now taken were carefully freed from adjoining liver tissue.

*Liver from rats fed azo dyes for* 2–12 *weeks.*—The findings, as illustrated in Fig. 4, agreed with those of other authors who used a high-protein diet as in the present work—for example, Price *et al.* (1952) and Striebich, Shelton and Schneider (1953). With 3′-Me-DAB (Fig. 4*a*), bile-duct hyperplasia was hardly evident at 17 days but became increasingly marked from 25 days to the latest time studied (80 days). Fibrosis was limited at 25 days but progressively increased, although even at 80 days the liver structure showed no serious damage. Some fat deposition was evident at 35 days. The right and right-median lobes did not differ from the other lobes in the time of appearance of the changes.

With 4′-F-DAB (Fig. 4*b*), bile-duct hyperplasia was slight even at 5 weeks. There being no evidence of leucocyte infiltration, the reason for the high DNA values reported below is obscure. At 7 weeks, however, both parenchymal and bile-duct hyperplasia was evident, with some fibrosis in one of the two rats studied ; neither rat showed serious liver damage. With 2-Me-DAB or 4′-Me-DAB the liver was of almost normal appearance even at 5 weeks (Fig. 4*c* and *d*).

### *Biochemical " Markers "*

Since " hepatomas " as used for biochemical studies may vary in nature, particularly from one laboratory to another, they warrant checking with respect to certain " marker " constituents (Potter, 1962 ; Reid, 1962*a*). One useful marker is glucose-6-phosphatase, which is almost absent from normal bile-duct cells, from many primary hepatomas, and from Novikoff hepatomas, but present in " Morris 5123 " transplanted hepatomas ; another is glucose-6-phosphate dehydrogenase, which is high in at least some primary hepatomas but normal in Morris 5123 hepatomas (see citations in Pitot, 1962, Reid, 1962*a*, and Morris, 1963). The DNA content, per g. of tissue, is also useful as a parameter for comparing results from different laboratories, particularly since some authors express their results on the basis of DNA rather than tissue weight.

*Glucose*-6-*phosphatase.*—The nodules were usually low in, but not devoid of, this enzyme (Table II). There were tendencies, not statistically significant, for the level to be particularly low in highly necrotic nodules and in adenocarcinomas as distinct from trabecular carcinomas. The levels in " large-celled " hepatomas were of the same order as that reported by Pitot (1960) for a large-celled hepatoma induced by 3′-Me-DAB, and some nodules with " hyperplasia and cholangioma " may have been counterparts of a " cholangio-carcinoma " nodule which he found to be almost devoid of activity. Precancerous liver was not studied, but according to the literature (Hadjiolov, 1962 ; Reid, 1962*a*) a decrease in activity is not consistently found within 4 weeks.

*Glucose*-6-*phosphate dehydrogenase.*—This enzyme (assayed during a collaborative study with Dr. P. McLean) showed the expected high activity in the hepatomas, irrespective of their histological appearance (Table II). High activity was likewise found in a hyperplastic nodule, which evidently differed from those studied by Emmelot, Bos, Brombacher and Hampe (1959). A rise was also found in precancerous liver (3′-Me-DAB or 4′-F-DAB), but only after four weeks. Kotnis, Narurkar

TABLE II.—*Glucose-6-phosphatase, Glucose-6-phosphate dehydrogenase, and DNA*

In this and subsequent Tables, the mean experimental values are tabulated relative to controls taken as = 100, all values having first been calculated as μmoles/g./min. (for enzymes) or mg./g. (for DNA). Values following the symbol ± represent standard errors. (In parentheses : number of observations and, where appropriate, the probability $P$ that the difference from controls could be due to chance.)

| | Glucose-6-phosphatase | Glucose-6-phosphate dehydrogenase | DNA* |
|---|---|---|---|
| Mean value in controls | 3·0 (=100) | 5·1 (=100) | See Fig. 5 (=100) |
| *Liver from rats fed 2-Me-DAB (virtually non-carcinogenic)* | | | |
| 7 days | | | 163(1) |
| 12–19 days | | | 28(2) } 51 ± 14 |
| 35–51 days | | 73 (1) | 75(2) } ($P$ < 0·05) |
| *Liver from rats fed 4′-Me-DAB (virtually non-carcinogenic)* | | | |
| 12–23 days | | | 110 (3) |
| 35–51 days | | 135 (1) | 101 (2) |
| *Liver from rats fed 3′-Me-DAB (highly carcinogenic)* | | | |
| 3 days | | | } See Fig. 5 |
| 7–20 days | | 106 (2) | } See Fig. 5† ; |
| 27–45 days | | 169 ± 34 (7 ; $P$ < 0·1) | } 144 ± 9 |
| 80 days | | | } (21 ; $P$ < 0·001) |
| *Liver from rats fed 4′-F-DAB (highly carcinogenic)* | | | } See Fig. 5 |
| 7 days | | 74 (3) | } See Fig. 5† ; |
| 12–27 days | | 195 ± 33 (5 ; $P$ < 0·05) | } 154 ± 19 |
| 35–51 days | | | } (11 ; $P$ < 0·025) |
| *Nodules from rats fed 3′-Me-DAB* | | | |
| Hepatoma nodules | 42 ± 13 (16 ; $P$ < 0·001) | 201 ± 34 (9 ; $P$ < 0·025) | 236 ± 21 (12 ; $P$ < 0·001) |
| *Hepatoma sub-categories :* | | | |
| Metastases | 4 (1) | 290 (1) | |
| Necrosis limited | 58 (7) | 226 (3) | 267 (5) |
| Necrosis very extensive | 28 (4) | 178 (4) | 240 (3) |
| Adenocarcinoma | 16 (3) | 243 (2) | |
| Trabecular carcinoma | 64 (8) | 198 (3) | 48 (8) |
| Mainly small-celled | | | 2247 (2) |
| Mainly large-celled | 29 (5) | 159 (2) | |
| Leucocytes abundant | 65 (3) | 360 (1) | 180 (3) |
| Hyperplastic nodules | 23 (8)‡ | 186 (1) | 149 (5)‡ |
| *Hepatoma and hyperplastic nodule sub-category :* | | | |
| Extensive fibrosis | 24 (6) | 215 (5) | 184 (10)† |

* Some of the values were kindly furnished by Mr. M. K. Turner.

† Where both left-lobe and right-lobe analyses were performed, the results are averaged for tabulation.

‡ Including some cholangioma-type nodules.

and Sahasrabudhe (1962*b*) found evidence of faster operation of the " hexose monophosphate shunt " (of which the first step is governed by glucose-6-phosphate dehydrogenase) in primary hepatomas but not in precancerous liver obtained by feeding with DAB. There is no other literature for short periods of azo-dye feeding.

*DNA*.—Table II further shows that the carcinogenic dyes markedly increase the amount of DNA per g., whereas 2-Me-DAB reduces the amount. The histological results offer no explanation of the changes found with 4′-F-DAB or 2-Me-DAB, which are in disagreement with published observations cited by Reid (1962*a*).

Moreover, the bile-duct proliferation found with 3′-Me-DAB fed more than 3 weeks does not account for the high values often seen—particularly in the right lobes—at 1–3 weeks (Fig. 5). There is a surprising lack of information in the literature concerning *early* effects of hepatocarcinogens on the content of DNA per

FIG. 5.—DNA in precancerous liver and nodules. At day " O " the rats were aged about 7 weeks ; in top portion, the oldest rats are on the right, and the DNA values are expressed in mg. (Note that the " amount/g. in controls " axis does not start at zero.)

A different symbol is used for each feeding series ; the symbols ▲ and ◇ refer respectively to August-strain rats and to Buffalo-strain rats, the hepatomas in the latter being " Morris 5123 " transplants (Reid and Morris, 1963). The symbols ▮▯ and ■ refer respectively to left-lobe and right-lobe values obtained in a single series ; the points shown ● are also right-lobe values, as is (for 4′-F-DAB but not for 3′-Me-DAB) the point shown ⊖. For other values no particular lobe was selected. The determinations represented by open symbols—including, in the case of nodules and the corresponding control tissue, half-filled circles—were made in unpublished experiments by Mr. M. K. Turner. The nodules are grouped into a left column representing hyperplastic nodules and choliangiomas, and other columns representing hepatomas with the least necrotic on the left and the most necrotic on the right.

g. of tissue or per nucleus. The early increase in DNA per g. now found with 3′-Me-DAB or 4′-F-DAB might be due to increased ploidy. Maini and Stich (1961) found increased ploidy at 8 weeks but, with a different analytical technique, Cunningham, Griffin and Luck (1950) and Price, Miller, Miller and Weber (1950) found no increase at 4–10 weeks. Conversely, the decrease found with 2-Me-DAB could be due partly to decreased ploidy, although ploidy is normal at 8 weeks (Maini and Stich, 1961). The high DNA values found, as expected, in the hepatomas (irrespective of their histology) may have been partly due to increased ploidy (Reid, 1962*a*).

The changes in DNA per g. as discussed above are expressed relative to controls which themselves showed a progressive fall in DNA per g. during the feeding period (Fig. 5), perhaps due to cytoplasmic hypertrophy (Iversen and Thamsen, 1956). In some control rats the trend was ultimately reversed (Fig. 5, top right). It is unlikely that the effects of 3′-Me-DAB and 4′-F-DAB on DNA are due to arrest of supposed hypertrophy, since the absolute weight of the liver was seldom lower than that in corresponding controls. However, the effect of 2-Me-DAB could be partly due to enhanced hypertrophy since liver weight tended to rise.

### *Acid-soluble Nucleotides*

The results are given in the form of tables, accompanied by graphs which show the results for 3′-Me-DAB in more detail together with results obtained for the transplanted hepatomas studied by Reid and Morris (1963). Comment on the latter results is made in the Discussion. In the tabulation of the results for precancerous liver, the different lobes of the liver have been treated as equivalent in view of the results shown in Fig. 6–11 ; in control rats the different lobes showed no differences and, moreover there were no marked differences between young and old controls (see Tables III–V). Results for the " normal " (precancerous ?) liver adjoining the hepatoma nodules are presented but not discussed.

*Uridine nucleotides.*—With each of the azo dyes, but particularly with 3′-Me-DAB, a marked rise in UMP was evident as early as one week after commencement of feeding (Table III and Fig. 6). Up to 3 weeks there was some correlation with carcinogenicity, the rise being smallest with the non-carcinogenic dyes ; but thereafter there was a marked rise with 4′-Me-DAB and only a moderate rise with 4′-F-DAB. The rise in UDP may well be unrelated to carcinogenicity, since 2-Me-DAB fed for up to 19 days tended to raise the level ; but the rise in UTP showed a clear relationship to carcinogenicity, particularly if only the first 3 weeks of feeding are considered. With 3′-Me-DAB the levels of UDP and UTP were almost back to normal after 30 days. The nodules ultimately obtained were usually somewhat high in UDP but not in UMP or UTP ; indeed, where there was marked fibrosis, the level of UTP was very low (Table III).

Table III also gives results for conjugated uridine nucleotides. With one exception—UDPglucuronate in rats fed 4′-F-DAB—increases were found at an early stage of feeding, whichever dye was given. After about 3 weeks, as is illustrated for 3′-Me-DAB (Fig. 7), the levels tended to fall towards normal. The early increases showed no correlation with carcinogenicity or in the case of UDP-glucuronate, an inverse correlation. The nodules eventually obtained showed almost normal levels of UDPacetylglucosamine but consistently low levels of UDPglucose and UDPglucuronate ; each of the three nucleotides was particularly low when fibrosis was prominent in the nodule (Table III, footnote).

*Adenosine avd guanosine nucleotides.*—In rats fed 3′-Me-DAB, AMP showed a biphasic change—an increase, followed by a decrease to sub-normal values (Table IV and Fig. 8). For ADP and, transiently, for ATP there was a small but significant decrease, with no prior increase. The decreases in AMP and ADP, but not that in ATP, show a correlation with carcinogencity. In the nodules induced by 3′-Me-DAB, ADP was consistently decreased, and ATP was usually decreased. ATP

Fig. 6.—Uridine-5′-mono-, di- and tri-phosphate in precancerous liver (3′-Me-DAB, for number of days indicated on axis), in nodules, and in liver adjoining nodules. (Note use of compressed scale for UMP.)

In Fig. 6–11 ◐ denotes left and left-median lobes, ◑ denotes right and right-median lobes, ● denotes liver taken without lobe selection, △ denotes "hyperplastic nodules" produced by 3′-Me-DAB, ▲ denotes hepatomas produced by 3′-Me-DAB and plotted with the least necrotic on the left and the most necrotic on the right, and ■ denotes Morris 5123 hepatomas (Reid and Morris, 1963). The symbols bear no relation to those used in Fig. 5.

TABLE III.—*Acid-soluble Nucleotides : Uridine Derivatives*

| | UMP | UDP | UTP | UDPglucose | UDPglucu-ronate | UDPacetyl-glucos-amine |
|---|---|---|---|---|---|---|
| Mean value in controls, µmoles/g.* | 0·14, 0·09 (=100) | 0·06, 0·05 (=100) | 0·11, 0·11 (=100) | 0·51, 0·60 (=100) | 0·41, 0·36 (=100) | 0·29, 0·35 (=100) |
| *Liver from rats fed 2-Me-DAB (virtually non-carcinogenic)* | | | | | | |
| 7–19 days | 118 (3) | 222 (3) | 123 (3) | 163 (3) | 142 (3) | 192 (3) |
| 35–39 days | 137 (2) | 99 (2) | 174 (2) | 130 (2) | 140 (2) | 147 (2) |
| | 126 ±9 (P< 0·05) | 7–35 : 193 ±37 (P<0·1) | 144 ±21 | 150 ±18 (P< 0·1) | 141 ±15 (P< 0·1) | 174 ±19 (P< 0·025 |
| *Liver from rats fed 4′-Me-DAB (virtually non-carcinogenic)* | | | | | | |
| 5–19 days | 109 (2) | 127 (2) | 78 (2) | 155 (3) | 131 (3) | 141 (3) |
| 24–39 days | 239 (3) | 108 (3) | 124 (3) | 126 (3) | 125 (3) | 115 (3) |
| 3 months, then 3 months off dye | 128 (1) | 202 (1) | 127 (1) | | 137 (1) | |
| | 177 ±33 (P< 0·1) | 128 ±20 | 125 ±21 | 140 ±18 (P< 0·1) | 129 ±11 (P< 0·05) | 128 ±15 |
| *Liver from rats fed 3′-Me-DAB (highly carcinogenic)* | | | | | | |
| 3–80 days : see Fig. 6 and 7 for complete time course | 3–80 days : 240±33 (18 ; P < 0·001) | 3–80 days : 152±13 (19 ; P < 0·005) | 7–24 days : 169±22 (9 ; P < 0·025) | 5–23 days : 140±10 (11 ; P < 0·005) | 7–24 days : 125±10 (10 ; P < 0·05) | 3–80 days 151±9 (20 ; P < 0·001) |
| *Liver from rats fed 4′-F-DAB (highly carcinogenic)* | | | | | | |
| 7–20 days | 137 (3) | 227 (3) | 197 (4) | 134 ±16 (5 ; P<0·1) | 95 (3) | 183 (4) |
| 27–39 days | 133 (3) | 178 (3) | 261 (3) | 105 (3) | 85 (3) | 158 (3) |
| | 135 ±7 (P< 0·005) | 202 ±25 (P< 0·01) | 223 ±42 (P< 0·05) | | 90 ±8 | 172 ±9 (P< 0·001) |
| *" Normal " liver adjoining nodules induced by 3′-Me-DAB* | | | | | | |
| | 187 (3) | 157 (3) | 73 (2) | 95 (3) | 83 (3) | 78 (3) |
| *Nodules induced by 3′-Me-DAB* | | | | | | |
| Hepatoma nodules | 118 ±10 (9; P < 0·1) | 138 ±10 (9; P < 0·01) | 81 ±19 (5) | 69 ±13 (9; P < 0·05) | 34 ±5 (9; P < 0·001) | 94 ±7 (9) |
| *Hepatoma sub-categories :* | | | | | | |
| Necrosis limited | 117 (6) | 152 (5) | 84 (4) | 63 (6) | 36 (6) | 97 (6) |
| Necrosis very extensive | 89 (2) | 132 (2) | | 44 (2) | 29 (2) | 76 (2) |
| Adenocarcinoma | 110 (1) | 156 (1) | 134 (1) | 93 (1) | 48 (1) | 114 (1) |
| Trabecular carcinoma | 143 (5) | 123 (5) | 71 (4) | 51 (5) | 18 (5) | 99 (5) |
| Mainly small-celled | 103 (3) | 134 (3) | 111 (1) | 58 (3) | 40 (3) | 91 (3) |
| Mainly large-celled | 82 (1) | 183 (1) | | 67 (1) | 17 (1) | 82 (1) |
| Hyperplastic nodules | 36 (1) | 133 (1) | 104 (1) | 31 (1) | 17 (1) | 67 (1) |
| *Hepatoma and hyperplastic nodule sub-category :* | | | | | | |
| Extensive fibrosis | 90 (4) | 119 (4) | 21 (2)† | 35 (4)† | 21 (4)† | 78 (4)† |

* In Tables III–V, two values are tabulated—for young controls, and for old controls (with which rats with nodules were compared), respectively.

† Comparison of the values (relative to controls) for nodules showing marked fibrosis with those for nodules showing little fibrosis gave the following values for difference of means ± s.e.:—UTP, 84 ±24 with D.F. (degrees of freedom) = 3 ($P \pm 0·05$) ; UDPglucose, 34 ±9 with D.F. = 8 ($P < 0·005$) ; UDPglucuronate, 19 ±9 with D.F. = 8 ($P < 0·1$) ; UDPacetylglucosamine, 26 ±9 with D.F. = 8 ($P < 0·025$).

TABLE IV.—*Acid-soluble Nucleotides : Adenosine and Guanosine Mononucleotides:*

| | AMP | ADP | ATP | GMP | GDP* | GTP |
|---|---|---|---|---|---|---|
| Mean value in controls, μmoles/g. | 0·79, 0·70 (=100) | 1·2, 1·2 (=100) | 0·88, 0·68 (=100) | 0·14, 0·12 (=100) | 0·26, 0·25 (=100) | 0·11, 0·08 (=100) |
| *Liver from rats fed 2-Me-DAB (virtually non-carcinogenic)* | | | | | | |
| 7–19 days | 81 (3) } 86 ±10 | 102 (3) } 102 ±7 | 179 (2) | 57 (3) | 82 (3) } 80 ±13 | 100 (1) |
| 25–39 days | 94 (2) } | 103 (2) } | 87 (2) | 127 (2) | 74 (1) } | 162 (2) |
| *Liver from rats fed 4′-Me-DAB (virtually non-carcinogenic)* | | | | | | |
| 5–19 days | 96 (3) } 94 ±12 | 98 (3) } 89 ±5 (P< 0·1) | 123 (3) | 36 (2) | 100 (1) | 20 (1) } 77 ±27 |
| 24–39 days | 91 (3) } | 85 (2) } | 93 (3) | 123 (3) } 134 ±16 | 102 (2) | 96 (3) } |
| 3 months, then 3 months off dye | 126 (1) | 68 (1) } | 170 (1) | 167 (1) } | | |
| *Liver from rats fed 3′-Me-DAB (highly carcinogenic)* | | | | | | |
| 3–80 days; see Fig. 8 and 9 for complete time course | 10–23 days: 139 ±16 (8; $P < 0·05$) 29–42 days: 61 ±6 (5; $P < 0·005$) | 7–80 days: 80 ±3·5 (20; $P <$ 0·005) | 10–24 davs: 72 ±8 (9; $P <$ 0·01) | No significant changes | 3–35 days: 78 ±1·5 (9; $P <$ 0·001) 80: 138 (1) | 3–10 days: 185 ±29 (6; $P < 0·05$) 29–80 days: 132 ±11 (4; $P < 0·1$) |
| *Liver from rats fed 4′-F-DAB (highly carcinogenic)* | | | | | | |
| 7–20 days | 73 (4) } 69 ±6 ($P <$ 0·005) | 70 (4) } 74 ±3 ($P <$ 0·001) | 138 (3) } 121 ±20 | 63 (2) } 7–35: 65 ±7 ($P <$ 0·025) | 58 (3) } 66 ±5 ($P <$ 0·005) | 92 (4) } 20–39: 181 ±15 ($P <$ 0·025) |
| 27–39 days | 63 (3) } | 79 (3) } | 104 (3) } | 83 (3) } | 77 (2) } | 191 (3) } |
| *"Normal" liver adjoining nodules induced by 3′-Me-DAB* | 123 (3) | 79 (3) | 66 (3) | 153 (3) | 125 (1) | 44 (1) |
| *Nodules induced by 3′-Me-DAB* | | | | | | |
| Hepatoma nodules | 76 ±10 (9; $P < 0·05$) | 40 ±6 (9; $P <$ 0·001) | 42 ±13 (9; $P <$ 0·005) | 109 ±12 (9) | 46 ±20 (4; $P <$ 0·1) | 49 ±20 (4; $P < 0·1$) |
| *Hepatoma sub-categories:* | | | | | | |
| Necrosis limited | 73 (6) | 43 (6) | 54 (6) | 105 (6) | 51 (3) | 62 (3) |
| Necrosis very extensive | 81 (2) | 26 (2) | 22 (2) | 86 (2) | | |
| Adenocarcinoma | 58 (1) | 58 (1) | 94 (1) | 113 (1) | | 68 (1) |
| Trabecular carcinoma | 85 (5) | 37 (5) | 24 (5) | 122 (5) | 46 (4) | 45 (3) |
| Mainly small-celled | 91 (3) | 45 (3) | 59 (3) | 112 (3) | 40 (2) | |
| Mainly large-celled | 61 (1) | 46 (1) | 22 (1) | 85 (1) | | |
| Hyperplastic nodules | 36 (1) | 23 (1) | 7 (1) | 54 (1) | | 7 (1) |
| *Hepatoma and hyperplastic nodule sub-category:* | | | | | | |
| Extensive fibrosis | 65 (4) | 69 (4) | 15 (4) | 71 (4)† | 7 (1) | 14 (2) |

* Only in some experiments did GDP and ADPribose-P separate ; changes as given in Table V for the *mixed* peak do appear to represent parallel decreases in the two components.

† Comparison of the values (relative to controls) for nodules showing marked fibrosis with those for nodules showing little fibrosis gave difference of means ±s.e. 54 ±19 with degrees of freedom = 8 ($P < 0·025$).

tended to be particularly low in trabecular carcinomas and in nodules with extensive necrosis or fibrosis, but these trends were not statistically significant.

A decrease in GMP was found with 4'-F-DAB (Table IV) but not with 3'-Me-DAB (Fig. 9). A decrease in GDP was found with either dye, but not with the non-carcinogenic dyes. On the other hand, GTP showed an increase which in the

FIG. 7.—Conjugated uridine nucleotides in precancerous liver (3'-Me-DAB), in nodules, and in liver adjoining nodules. See Legend to Fig. 6.

case of 3'-Me-DAB may have been biphasic (Fig. 9), and in the case of 4'-F-DAB was not evident until 4 weeks ; there appeared to be a fair correlation with carcinogenicity. In the nodules induced by 3'-Me-DAB, GDP and GTP tended to be low, particularly if there were fibrosis. GMP likewise showed somewhat decreased values in fibrotic nodules as compared with non-fibrotic nodules (Table IV, footnote), the latter having normal or high levels of GMP. Comparison of Fig. 8

and Fig. 9, with respect to the nodules and to the precancerous liver obtained by giving 3′-Me-DAB for more than 3 weeks, shows trends of similarity between AMP and GMP, between ADP and GDP, and between ATP and GTP.

*Inosine monophosphate and "pyridine" nucleotides.*—As is evident from Table V and Fig. 10, IMP was decreased in precancerous liver, even with one week of

FIG. 8.—Adenosine-5′-mono-, di- and tri-phosphate in precancerous liver (3′-Me-DAB), in nodules, and in liver adjoining nodules. See Legend to Fig. 6.

treatment ; by two criteria—the magnitude and the duration of the decrease—the effect was more striking than that of the non-carcinogenic dyes. The level of IMP was consistently low in the nodules.

Table V and Fig. 10 also show that for the unidentified compound " post-AMP " there was an elevation, unrelated to carcinogenicity, in rats fed 3′-Me-DAB, but not in the nodules.

Values for " pyridine " nucleotides are shown in Table V and Fig. 11. During azo-dye feeding there was no consistent change in the level of NAD ; but the level was low in some of the nodules examined, particularly if there were fibrosis. Soon

TABLE V.—*Acid-soluble Nucleotides: Inosine-5′-monophosphate, Nicotinamide-ribose-Adenosine Dinucleotides, and "Post-AMP"*

| | IMP | NAD | NADP* | ADP-ribose-P +GDP | ADP-ribose-P (=$NADPH_2$)* | "Post-AMP" |
|---|---|---|---|---|---|---|
| Mean value in controls, µmoles/g. or, where so stated, $E_{260}$u./g. | 0·31, 0·29 (=100) | 0·44, 0·42 (=100) | 0·08, 0·095 (=100) | $E_{260}$ u.: 7·1, 6·9 (=100) | 0·3, 0·3 (=100) | $E_{260}$ u.: 3·6, 2·2 (=100) |
| *Liver from rats fed 2-Me-DAB (virtually non-carcinogenic)* | | | | | | |
| 7–19 days | 54 (3) | 77 (3) | 105 (2) | 107 (3) | 126 (3) | 169 (3) |
| 35–39 days | 120 (2) | 119 (1) | 110 (2) | 135 (2) | 117 (1) | 95 (2) |
| (combined) | | | 108 ±5 | 118 ±12 | 124 ±5 ($P<$ 0·025) | 7–35: 162 ±23 ($P<$ 0·1) |
| *Liver from rats fed 4′-Me-DAB (virtually non-carcinogenic)* | | | | | | |
| 5–19 days | 89 (2) | 63 (3) | 138 (3) | 109 (2) | 125 (1) | 100 (1) |
| 24–39 days | 77 (3) | 112 (3) | 124 (3) | 91 (3) | 84 (2) | 68 (3) |
| 3 months, then 3 months off dye | 77 (1) | 93 (1) | 126 (1) | 55 (1) | | |
| (combined) | 81 ±5 ($P<$ 0·025) | | 130 ±24 | 82 ±10 | | |
| *Liver from rats fed 3′-Me-DAB (highly carcinogenic)* | | | | | | |
| 3–80 days; see Fig. 10 and 11 for complete time course | 3–80 days: 71 ±9 (19; $P<0·005$) | No significant changes | 7–80 days: 61 ±4 (12; $P<0·001$) | 3–38 days: 75 ±4 (17; $P<0·001$) | 3–80 days: 73 ±4 (13; $P<$ 0·005) | 5–80 days: 195 ±27 (15; $P<$ 0·005) |
| *Liver from rats fed 4′-F-DAB (highly carcinogenic)* | | | | | | |
| 7–20 days | 34 (3) | 94 (4) | 54 (2) | 70 (4) | 66 (3) | 166 (1) |
| 27–39 days | 54 (3) | 117 (3) | 44 (3) | 80 (3) | 71 (2) | 121 (3) |
| (combined) | 44 ±8 ($P<$ 0·001) | | 48 ±6 ($P<$ 0·001) | 74 ±8 ($P<$ 0·025) | 68 ±8 ($P<$ 0·025) | 132 ±28 |
| *"Normal" liver adjoining nodules induced by 3′-Me-DAB* | 77 (3) | 77 (3) | 107 (3) | 80 (3) | 55 (1) | |
| *Nodules induced by 3′-Me-DAB* | | | | | | |
| Hepatoma nodules | 32 ±8 (8; $P<0·001$) | 56 ±11 (8; $P<0·005$) | 45 ±15 (8; $P<0·01$) | 44 ±12 (9; $P<0·005$) | 14 ±4 (4; $P<$ 0·001) | 105 ±29 (4) |
| *Hepatoma sub-categories:* | | | | | | |
| Necrosis limited | 28 (5) | 72 (5) | 55 (6) | 52 (6) | 17 (2) | 79 (3) |
| Necrosis very extensive | 17 (2) | 30 (2) | <15 (1) | 43 (2) | | |
| Adenocarcinoma | 39 (1) | 117 (1) | 155 (1) | 55 (1) | | |
| Trabecular carcinoma | 40 (4) | 49 (5) | <20 (5) | 56 (5) | <40 (4) | 102 (3) |
| Mainly small-celled | 27 (3) | 56 (2) | <39 (3) | 81 (3) | <15 (1) | 58 (1) |
| Mainly large-celled | 18 (1) | 40 (1) | <15 (1) | 22 (1) | | 115 (1) |
| Hyperplastic nodules | 17 (1) | 14 (1) | <15 (1) | 7 (1) | | |
| *Hepatoma and hyperplastic nodule sub-category:* | | | | | | |
| Extensive fibrosis | 51 (3) | 32 (4)† | 15 (3) | 28 (4) | 10 (1) | |

* The levels in some of the nodules studied were too low to measure, but have been taken as 15 (controls = 100) for the purpose of statistical calculation.

† Comparison of the values (relative to controls) for nodules showing marked fibrosis with those for nodules showing little fibrosis gave difference of means ±s.e. 37 ±18 with degrees of freedom =7 ($P<0·1$).

after the start of azo-dye feeding, NADP and $NADPH_2$ showed decreases (possibly preceded by an increase in the case of NADP) which were well correlated with carcinogenicity. In the nodules other than one adenocarcinoma, NADP was low—perhaps particularly low when there was marked necrosis or fibrosis—and $NADPH_2$ was very low.

FIG. 9.—Guanosine-5′-mono, di- and tri-phosphate in precancerous liver (3′-Me-DAB), in nodules, and in liver adjoining nodules. See Legend to Fig. 6.

According to some reports, the concentrations of the pyridine nucleotides in normal rat liver change with age. Mondy, Strength, Gray and Daniel (1954) found a rise with age in NAD. Caiger, Morton, Filsell and Jarrett (1962) found higher levels of liver $NADPH_2$ in mature rats than in young rats—as also found for aorta and lens tissue (P. Mandel, personal communication); but the data of Kotnis *et al.* (1962*b*) suggest a decrease with age in NAD + NADP and more especially in $NADH_2$ + $NADPH_2$. As is evident from the mean values given for controls in

Table V (the first value in each pair being for rats aged 8–12 weeks and the second for rats aged 6–15 months), no age effects have now been observed.

*Nucleotides in mouse hepatomas.*—Since nucleotide levels in normal rats have shown no marked alteration with age, the levels in the spontaneous mouse hepatomas now examined (Table VI) are best compared with those in liver from young mice of the same strain, rather than with the values (as also tabulated) for liver which, being from old mice in which hepatomas had not yet developed, may have been precancerous. Like the primary hepatomas induced in rats, the mouse hepatomas were low in UDPglucuronate, ADP, IMP, and $NADPH_2$. Moreover,

FIG. 10.—Inosine-5′-monophosphate and " Post-AMP " (an unidentified compound, see Experimental section) in precancerous liver (3′-Me-DAB), in nodules, and in liver adjoining nodules. See Legend to Fig. 6.

like the fibrotic primary hepatomas studied in rats, the mouse hepatomas were low in UTP, GMP and NAD, although fibrosis is not a prominent feature of the mouse hepatomas (Connell and Alexander, 1959). In contrast with the rat hepatomas, the mouse hepatomas were low in UMP and AMP and showed no increase in UDP or decrease in UDPglucose or NADP.

*Precancerous liver from adrenalectomized or thyroxine-treated rats.*—In rats given no azo dye, adrenalectomy causes a fall in UDPglucuronate and NADP and a depression (preceded by a rise) in UMP and ATP, whereas thyroxine treatment depresses the levels of UDPglucose, UDPacetylglucosamine, pyridine nucleotides (cf. Glock and McLean, 1955, and Knox, Auerbach and Lin, 1956) and all triphosphates but tends to raise the levels of monophosphates (Reid, 1961). The effects of these hormonal treatments in rats fed an azo dye warranted investigation from two points of view. Since there are reports that hepatocarcinogenesis may be retarded by adrenalectomy and accelerated by hyperthyroidism (Reid, 1962*a*), any reduction by adrenalectomy or enhancement by thyroxine of an azo-dye effect (termed " situation *1* " below) would argue that the effect may be important for hepatocarcinogenesis. On the other hand, if with dye feeding an effect of adrenalectomy or thyroxine that is normally demonstrable (in rats given no azo dye) is obliterated, or completely masked by an effect of the azo dye itself, this result (" situation *2* ") would suggest that azo-dye treatment can over-ride other influ-

FIG. 11.—Nicotinamide-adenine nucleotides (" pyridine nucleotides ") in precancerous liver (3′-Me-DAB), in nodules, and in liver adjoining nodules. See Legend to Fig. 6.

ences, although it would not prove identity in primary site of action between azo-dyes and hormones. There is already some evidence (cited by Reid, 1962*a*) that " induction " of certain enzymes can be blocked by azo-dye feeding, and Knox *et al.* (1956) have collated other examples of metabolic situations where one influence over-rides another.

The results given in Table VI, (*b*) and (*c*), may now be examined in relation to the effects of 3′-Me-DAB as summarized in Tables III–V, firstly to ascertain if " situation *1* " applies to any of the nucleotide changes. Since the rise in UMP

TABLE VI.—*Acid-soluble Nucleotides (a) in Spontaneous Mouse Hepatomas, (b) in Adrenalectomized rats fed 3′-Me-DAB, and (c) in Rats fed 3′-Me-DAB and injected with Thyroxine*

The values—each of which represents a single experiment—are expressed as per cent of " controls ", namely (a) 4-month old mice of the same strain, (b) and (c) rats not adrenalectomized or given thyroxine but given 3′-Me-DAB (or, in parentheses, controls given *no* treatment). The injections of L-thyroxine (20 μg.) were given subcutaneously, once daily.

| | UMP | UDP | UTP | UDP-glucose | UDP-glucuronate | UDP-acetylglucosamine | AMP | ADP | ATP | GMP | GDP | GTP | IMP | NAD | NADP | $NADPH_2$ | " Post-AMP" |
|---|---|---|---|---|---|---|---|---|---|---|---|---|---|---|---|---|---|
| *(a) Mouse tissues* | | | | | | | | | | | | | | | | | |
| Control value, liver from 4-month mice, μmoles/g. | 0·115 | 0·059 | 0·185 | 0·50 | 0·42 | 0·24 | 0·56 | 1·20 | 1·40 | 0·085 | 0·14 | 0·21 | 0·11 | 0·71 | 0·115 | 0·11 | 4·3* |
| | (=100) | (=100) | (=100) | (=100) | (=100) | (=100) | (=100) | (=100) | (=100) | (=100) | (=100) | (=100) | (=100) | (=100) | (=100) | (=100) | (=100) |
| 14-month mice, liver without hepatomas | 98 | 90 | 44 | 125 | 81 | 155 | 154 | 117 | 96 | 151 | 150 | 76 | 88 | 77 | 110 | 165 | 126 |
| Hepatomas . . . | 36 | 85 | 49 | 120 | 50 | 140 | 60 | 60 | 102 | 40 | 85 | 116 | 29 | 38 | 90 | 53 | 81 |
| *(b) Adrenalectomized rats, precancerous liver* | | | | | | | | | | | | | | | | | |
| Adrenalectomy, day −1; 3′-Me-DAB, days 1–5 | 68 | 56 | 50 | 72 | .. | 62 | 74 | .. | 95 | 67 | 108 | 66 | .. | .. | 125 | .. | .. |
| | (96) | (65) | (49) | (121) | .. | (94) | (55) | .. | (104) | (53) | (48) | (128) | .. | .. | (285) | .. | .. |
| Adrenalectomy, day −1; 3′-Me-DAB, days 1–21 | 49 | 153 | .. | 235 | 80 | 164 | 78 | 81 | 138 | 76 | 85 | .. | .. | 78 | 156 | 124 | .. |
| | (146) | (175) | .. | (270) | (94) | (220) | (162) | (99) | (130) | (130) | (121) | .. | .. | (114) | (101) | (155) | .. |
| Adrenalectomy, day −1; 3′-Me-DAB, days 1–23 | 88 | 63 | 45 | 99 | 59 | 72 | 80 | 114 | 91 | 158 | 90 | 108 | .. | 60 | 112 | 122 | 75 |
| | (170) | (92) | (85) | (93) | (70) | (87) | (82) | (94) | (76) | (142) | .. | (62) | .. | (96) | (60) | .. | (230) |
| *(c) Thyroxine-treated rats, precancerous liver* | | | | | | | | | | | | | | | | | |
| Thyroxine, days 1–6; 3′-Me-DAB, days 1–5 | 315 | 68 | 58 | 48 | 59 | 88 | 92 | 96 | 66 | .. | .. | 44 | .. | .. | .. | .. | <20 |
| | (440) | (79) | (57) | (81) | (52) | (132) | (68) | (96) | (72) | .. | .. | (85) | .. | .. | .. | .. | (<30) |
| Thyroxine, days 1–21; 3′-Me-DAB, days 1–21 | 73 | 198 | 182 | 198 | 160 | 225 | 51 | 62 | 86 | 75 | .. | .. | 47 | .. | 140 | .. | .. |
| | (213) | (225) | (198) | (227) | (195) | (300) | (106) | (76) | (80) | (128) | .. | .. | (105) | .. | (91) | .. | .. |
| Thyroxine, days 1–24; 3′-Me-DAB, days 1–23 | 166 | 65 | 61 | 44 | 44 | 67 | 93 | 91 | 65 | 120 | .. | 61 | 119 | 64 | 128 | .. | <10 |
| | (320) | (93) | (48) | (42) | (52) | (81) | (98) | (75) | (55) | (107) | .. | (35) | (72) | (104) | (69) | .. | (<25) |

* $E_{260}$ u./g., not μmoles/g.

with dye feeding was reduced by adrenalectomy and, in 2 out of 3 experiments, was enhanced by thyroxine, this situation indeed holds for UMP It likewise holds for UTP, UDPglucuronate, NADP and $NADPH_2$ as judged by the effect of adrenalectomy, but not as judged by the effect of thyroxine—for which the results actually showed the converse of situation *1* in the case of UTP and NADP and also of UDPglucose. On the other hand, situation *1* fits the thyroxine results for ATP and " post-AMP " but not the adrenalectomy results for ATP, and in the case of ADP both sets of results are contrary to situation *1*. It appears, then, that only the dye-induced rise in UMP and, possibly, the rises in UDPglucuronate and " post-AMP " and the fall in $NADPH_2$ fit situation *1* and could, therefore, by the present criterion rank as important events in azo-dye carcinogenesis.

The results of Table VI may now be examined in relation to "situation *2*". In no case does this situation consistently hold both for adrenalectomy and for thyroxine treatment. The expected effects of adrenalectomy, superimposed on the azo-dye effects, were in fact observed for UMP and UDPglucuronate, although not for ATP, NADP and perhaps GTP. Similarly, the expected effects of thyroxine were observed for ATP and NADP and also, in 2 out of 3 experiments, for UMP, UTP, UDPglucose and UDPacetylglucosamine, but not for AMP. It is concluded that only for AMP, ATP and NADP is there some evidence that azo-dye feeding can swamp hormonal effects on nucleotide levels.

### *Ribonucleases and Phosphodiesterases*

As was pointed out in the Introduction, liver cytoplasm contains at least two ribonucleases, neither of which acts on the cyclic mononucleotides produced by their attack in RNA ; the opening up of the phosphate bridge in these nucleotides is effected by distinct phosphodiesterases, possibly different for each nucleotide.

*Alkaline ribonuclease.*—The effect of 3′-Me-DAB in lowering the alkaline-ribonuclease activity of microsomes, as shown by Reid and Lotz (1958) and by Hadjiolov (1962), is evidently transient and unrelated to carcinogenicity (Table VII). Moreover, the " total " activity found in supernatant fractions after destruction of the endogenous inhibitor is normal in primary hepatomas whatever their histological appearance, although possibly increased in hyperplastic nodules (Table VII).

*Acid ribonuclease.*—With the further experiments now performed, the results for this enzyme are more clear-cut than those of Reid and Lotz (1958). The "total" activity found in mitochondrial fractions (containing lysosomes) after freezing and thawing is somewhat diminished in nodules induced by 3′-Me-DAB (Table VII) ; this finding is compatible with other reports (Reid, 1962*a*) except that of Ledoux, Pileri and Vanderhage (1958) who found increased activity in homogenates of DAB hepatomas. On the other hand, the activity found in supernatant fractions—normally low—is strikingly increased both in precancerous liver—even at 5 days—and in nodules other than small-celled hepatomas. The tendency for the activity to be particularly high in nodules with marked necrosis may well have been due to chance (Table VII, footnote). Although the feeding of non-carcinogenic azo dyes tended to give some increase in supernatant-fraction activity, the extent of the increase is clearly correlated with carcinogenicity.

*Phosphodiesterases.*—The assay of different cell fractions for activity towards cyclic mononucleotides (nucleoside cyclic phosphates) has disclosed few changes in precancerous liver or hepatomas (Table VIII). With 2′,3′-AMP as substrate, the activity in microsomal fractions—which is normally low—showed in precancerous liver a slight rise which may be related to carcinogenicity, and in nodules a fall which was perhaps greater in hepatomas than in hyperplastic nodules. Mitochondrial fractions, which normally have low activity towards 2′,3′-CMP, showed even lower activity in the case of nodules induced by 3′-Me-DAB. Microsomal fractions, which normally have very low activity towards 2′,3′-UMP, showed somewhat increased activity in rats fed 3′-Me-DAB for more than 14 days ; but no increase was found with 4′-F-DAB.

TABLE VII.—*Ribonucleases*

| | Alkaline ribonuclease, microsomal fraction | Alkaline ribonuclease, supernatant fraction | Acid ribonuclease, mitochondrial fraction | Acid ribonuclease, supernatant fraction |
|---|---|---|---|---|
| Mean value in controls, $\mu$ moles (calculated as mononucleotide) / g. / min. | 0·04 (= 100) | 0·17 (= 100) | 1·7 (= 100) | 0·35 (= 100) |
| *Liver from rats fed 2-Me-DAB (virtually non-carcinogenic)* | | | | |
| 19 days . . . | 95 (1) | | | 110 (1) } 120 ±26 |
| 35–39 days . . . | 87 (1) | | | 123 (3) } |
| *Liver from rats fed 4′-Me-DAB (virtually non-carcinogenic)* | | | | |
| 19 days . . . | 114 (1) | | | 136 (1) } 135 ±7 ($P<0·025$) |
| 24–39 days . . . | 96 (1) | | | 138 (3) } |
| *Liver from rats fed 3′-Me-DAB (highly carcinogenic)* | | | | |
| 5–17 days . . . | | | | 146 (5) } 170 ±16 ($P<0·001$) |
| 20–28 days . . . | 54 ± 14 (5 ; $P<0·05$) | No change (Reid and Lotz, 1958) | No change (Reid and Lotz, 1958) | 174 (7) } |
| 35–39 days . . . | 109 (3) | | | 202 (3) } |
| *Liver from rats fed 4′-F-DAB (highly carcinogenic)* | | | | |
| 19 days . . . | 114 (1) | | | 195 (1) } 339 ±46 ($P<0·005$) |
| 27–51 days . . . | 116 (1) | 88 (2) | | 367 (5) } |
| *Nodules from rats fed 3′Me-DAB* | | | | |
| Hepatoma nodules . | No change (Reid and Lotz, 1958) | 101±12 (10) | 70±6 (11 ; $P<0·001$) | 197±20 (19 ; $P<0·001$) |
| *Hepatoma sub-categories :* | | | | |
| Metastases . . | | | | 231 (2) |
| Necrosis limited . | | 87 (7) | 75 (6) | 156 (9) } * |
| Necrosis very extensive | | 125 (1) | 52 (3) | 230 (5) } |
| Adenocarcinoma . | | 88 (5) | 60 (3) | 159 (6) |
| Trabecular carcinoma | | 112 (4) | 83 (6) | 204 (9) |
| Mainly small-celled . | | 103 (4) | 92 (3) | 102 (4) } † |
| Mainly large-celled . | | 98 (2) | 80 (2) | 231 (5) } |
| Leucocytes abundant | | 92 (1) | 68 (1) | 190 (2) |
| Hyperplastic nodules . | | 218 (1) | 85 (2) | 284 (3) |
| *Hepatoma and hyperplastic nodule sub-category :* | | | | |
| Extensive fibrosis . | | 116 (5) | 66 (8) | 226 (13) |

* Standard error of difference of means = ±46 ($P<0·2$)
† Standard error of difference of means = ±54 ($P<0·05$)

TABLE VIII.—*Nucleoside Cyclic Phosphate-diesterases*

| | Substrate | Whole cytoplasm | Mitochondrial fraction | Microsomal fraction | Supernatant fraction |
|---|---|---|---|---|---|
| Mean value in controls, | 2′,3′-AMP | 4·0 | 1·55 | 0·7 | 1·65 |
| $\mu$ moles / g. / min. | 2′,3′-GMP | 4·1 | 1·7 | | 2·4 |
| (= 100) | 2′,3′-CMP | 1·95 | 0·6 | 0·3 | 1·35 |
| | 2′,3′-UMP | 1·3 | 0·4 | 0·2 | 0·5 |
| | *Liver from rats fed 4′-Me-DAB (virtually non-carcinogenic)* | | | | |
| 25 days . . . | 2′,3′-AMP | 102 (1) | 73 (1) | 105 (1) | 113 (1) |
| 51 days . . . | 2′,3′-AMP | | | 105 (1) | |
| 25 days . . . | 2′,3′-CMP | 99 (1) | 90 (1) | 61 (1) | 114 (1) |
| 35 days . . . | 2′,3′-UMP | | | 55 (1) | |
| | *Liver from rats fed 3′-Me-DAB (highly carcinogenic)* | | | | |
| 13 days . . . | 2′,3′-AMP | | | 130 (2) } 123 | |
| 21–51 days . . | 2′,3′-AMP | $108 \pm 4$ (4) | $110 \pm 15$ (4) | 120 (4) } $\pm 6$ | $107 \pm 8$ (4) |
| | | | | } ($P < 0·01$) | |
| 27 days . . . | 2′,3′-GMP | | | | 96 (1) |
| 21–25 days . . | 2′,3′-CMP | $97 \pm 3$ (4) | $111 \pm 7$ (4) | $90 \pm 8$ (4) | $95 \pm 6$ (4) |
| 14 days . . . | 2′,3′-UMP | | | 80 (2) | |
| 19–35 days . . | 2′,3′-UMP | 102 (2) | 115 (2) | $115 \pm 19$ (5 ; $P < 0·05$) | 96 (3) |
| | *Liver from rats fed 4′-F-DAB (highly carcinogenic)* | | | | |
| 51 days . . . | 2′,3′-AMP | | | 215 (2) | |
| 27 days . . . | 2′,3′-GMP | | | | 215 (2) |
| 27–35 days . . | 2′,3′-UMP | | | 99 (2) | 106 (2) |
| | *Nodules from rats fed 3′-Me-DAB** | | | | |
| Hepatoma nodules . | 2′,3′-AMP | 95 (2) | $91 \pm 4$ (4) | $55 \pm 8$ (4 ; $P < 0·025$) | $110 \pm 4$ (5; $P < 0·1$) |
| | 2′,3′-GMP | 100 (1) | 107 (3) | | $101 \pm 6$ (4) |
| | 2′,3′-CMP | 88 (2) | $64 \pm 9$ (5 ; $P < 0·025$) | 97 (1) | $97 \pm 4$ (4) |
| | 2′,3′-UMP | 103 (1) | 99 (3) | $132 \pm 27$ (5) | $100 \pm 6$ (4) |
| Hyperplastic nodules . | 2′,3′-AMP | 105 (2) | 110 (2) | 82 (2) | 97 (2) |
| | 2′,3′-CMP | 85 (2) | 74 (2) | 80 (2) | 123 (2) |

* The hepatomas were mostly trabecular carcinomas ; there were no tendencies for the results to be influenced by histological features such as necrosis or fibrosis.

### DISCUSSION

It may first be emphasized that the analyses on the nodules showed few correlations between the extent of the biochemical changes and the histological character of the samples. With marked fibrosis there were particularly low levels of UTP, UDPglucose, UDPacetylglucosamine, GMP, and possibly UDPglucuronate, GDP, GTP, NAD and NADP. As discussed in a paper to follow (Reid, 1964*b*), these trends are not surprising since the formation of fibrous tissue implies faster utilization of at least some of these nucleotides. Hepatomas (or, more strictly, hepatocarcinomas) of the " small-celled " type were exceptional in one respect, that the activity of acid ribonuclease in the supernatant fraction was not consistently increased. Otherwise the results were apparently independent of histological character.

In nodules which contained areas of recognizable hepatoma tissue, but were highly necrotic elsewhere, the content of nucleotides (except, perhaps, ATP and NADP) was as high as in non-necrotic nodules and sometimes even as high as in normal liver. Moreover, " necrotic " nodules were not particularly low in anabolic enzymes or high in catabolic enzymes (Reid, 1964*b*). Beaufay, Van Campenhout and de Duve (1959) found that in liver with the pedicle ligated there was massive necrosis associated with a rise in the acid-ribonuclease activity of the supernatant fraction. However, the elevation in supernatant-fraction acid ribonuclease now reported was not particularly associated with necrosis, in contrast with the ribonuclease activity (presumably " total " cellular activity, and perhaps due to alkaline as well as to acid ribonuclease) studied histochemically by Amano and Daoust (1961) in precancerous liver and hepatomas. It appears, then, that " necrotic " areas in hepatomas are functionally similar to " healthy " areas. Goldacre and Sylvén (1962) have in fact shown that necrotic areas in tumours do contain at least some viable cells.

It further appears that " hyperplastic nodules " resemble hepatomas in respect of the parameters dealt with here and in the papers to follow (Reid, 1964*a*, 1964*b*). The preferred interpretation of this conclusion is not that " hepatomas " contain many normal cells—which, in a large nodule, seems unlikely—but that the hyperplastic nodules are already destined to become malignant, perhaps through a further biochemical change which, from observations by Emmelot, Bos, Brombacher and Hampe (1959), may lie in the area of energy supply.

The study of transplanted as distinct from primary hepatomas can be helpful for deciding what are the *minimum* biochemical requirements for malignancy, but transplantation can itself lead to variations in histological and biochemical characteristics (Pitot, 1962). The histological heterogeneity of primary hepatomas evidently does not seriously detract from their usefulness, although generalisations about hepatomas cannot be made from the present restricted study.

*Relevance of the nucleotide findings to azo-dye carcinogenesis.*—The preliminary results reported by Reid and Lotz (1958) have been largely confirmed by the present results, a summary of which is given in Fig. 2 of a subsequent paper (Reid, 1964*b*). The values now obtained for 80 days' feeding with 3′-Me-DAB will not be discussed, since they merely support the view (Reid, 1962*a*, 1964*b*) that following the initial insult of azo-dye feeding, most of the liver cells may become adapted or even " over-adapted "; these values show only one marked difference—the lack of a fall in NADP—from values reported by Wrba, Schönenberger, Bamann and Lang (1961) for rats fed DAB for 68 days.

All uridine nucleotides were increased in liver from rats fed 3′-Me-DAB, at least up to 24 days ; but only for UMP and UTP did this early increase show some correlation with the carcinogenicity of the dye. Indeed, the rise in UDPglucuronate was greatest with the non-carcinogenic dyes—a finding which outweighs the suggestion from the hormonal studies that this rise may be an important event in azo-dye carcinogenesis. The hormonal studies did, however, strongly support the view that the rise in UMP is important. It is suggested elsewhere (Reid, 1964*b*) that the rise in UMP is due to faster synthesis, perhaps accompanied by decreased phosphorylation to UDP and UTP because of the fall in ATP. Unlike 3′-Me-DAB, 4′-F-DAB gave a bigger elevation in UDP and UTP than in UMP and gave no fall in ATP.

The nodules ultimately obtained in rats fed 3′-Me-DAB showed high values only for UDP. UDPglucose, UDPglucuronate, and sometimes UDPacetylglucosamine and UTP were depleted, probably because of fibrous-tissue formation as discussed above but perhaps also because of faster synthesis of serum mucoprotein (Wada, Ohara, Sasaki, Nakajimo and Yachi, 1957). However, uridine nucleotides considered as a whole were little depleted, perhaps because faster utilization in the primary hepatomas is balanced by faster synthesis (Reid, 1964*b*).

Purine nucleotides showed a general trend towards low levels, particularly in the hepatoma nodules. However, in precancerous liver GMP was normal, and the decrease in AMP was preceded, when 3′-Me-DAB was used, by an increase which was lacking when 4′-F-DAB was used ; in the nodules both AMP and GMP were almost normal. Moreover, in precancerous liver ATP showed only a small decrease, of doubtful relevance to carcinogenesis, and GTP showed an increase which may well be relevant; in some of the nodules ATP and GTP were only slightly decreased. ADP, GDP and IMP did, however, consistently show decreases in precancerous liver and hepatomas, the early decreases (and the later decrease in AMP) being well correlated with the carcinogenicity of the dye. Since IMP is the precursor of both adenosine and guanosine nucleotides, and since the decrease in IMP was both early in onset and striking in extent, it would appear that the trend towards a fall in guanosine and especially adenosine nucleotides can be attributed to lack of IMP, probably due to impaired synthesis although no direct evidence of this is available. Impaired synthesis of IMP might, from the work of Henderson (1962) with ascites cells, be a consequence of " feedback inhibition " by adenine or adenosine, the levels of which in precancerous liver are unknown. Nucleotides such as ADP, ATP, and GMP may themselves exert feedback control on the synthesis of IMP (Wyngaarden and Ashton, 1959) and on the conversion of IMP into GMP (Magasanik and Karibian, 1960) ; but the balance of these controls is probably little altered in hepatocarcinogenesis since the changes in the levels of ADP, ATP and GMP are not dramatic.

The data for nicotinamide-adenine (" pyridine ") nucleotides show a trend of decrease, the early decreases in NADP and $NADPH_2$ being well correlated with carcinogenicity. However, there were almost normal levels of NAD in precancerous liver, as also found by Jedeikin, Thomas and Weinhouse (1956). Kotnis *et al.* (1926*b*) reported small decreases in NAD + NADP and in $NADH_2$ + $NADPH_2$ in rats fed DAB for $1\frac{1}{2}$ months or longer, but the lack of data for *individual* puridine nucleotides detracts from the value of this and certain other reports. NAD was likewise almost normal in some of the hepatomas, as in hepatomas studied by Jedeikin *et al.* (1956) and in cholangiomas studied by Glock and McLean (1957), but $NADPH_2$ consistently showed a marked decrease as also found by Glock and McLean (1957).

The suggestion that NAD synthesis is impaired in carcinogenesis (Morton, 1958) lacks adequate support, although Kotnis *et al.* (1962*a*) found that the effect of nicotinamide injections in elevating *total* pyridine nucleotides was impaired by treatment with DAB. Since NAD is the precursor of the other pyridine nucleotides, the decrease now observed in NADP and $NADPH_2$ but not in NAD could hardly be due to decreased NAD synthesis, although it could be due to impaired conversion of NAD into NADP. No ready explanation can be given of the tendency, in tumours induced by azo dyes, for $NADPH_2$ to decrease more than NADP. Transhydrogenase is low in such tumours (Reynafarje and Potter, 1957), but it is uncertain whether the transhydrogenase pathway is important in the formation (from NADP and $NADH_2$) of $NADPH_2$ as distinct from its re-conversion into NADP. There is evidence in the literature (Reynafarje and Potter, 1957 ; see also Reid, 1962*a*) for loss of $NADPH_2$-cytochrome c reductase, but this loss would favour accumulation rather than lowering of $NADPH_2$. The latter situation would likewise be expected if the " HMP shunt " were accelerated, such an acceleration being suggested by the present finding of increased glucose-6-phosphate dehydrogenase activity and by various published observations (Emmelot *et al.*, 1959 ; Chayen, Bitensky, Aves, Jones, Silcox and Cunningham, 1962 ; Kotnis *et al.*, 1962*b*).

*Are any if the nucleotide findings generally applicable to hepatocarcinogenesis ?*— Several workers have studied effects of feeding ethionine, although apparently they did not check whether the feeding conditions were such as to result eventually in liver tumours. Even with brief feeding, Caldarera, Budini, Barbiroli and Rabbi (1962) found decreases in purine nucleotides other than AMP and GMP, essentially as now found with azo-dye feeding, but in contrast with the present findings there appeared to be decreases in NAD, UMP and UTP and little change in other uridine nucleotides. Their values for UDP and possibly other uridine nucleotides might, however, be unreliable since re-chromatography was not performed. Other workers have likewise found that ethionine decreases ATP (Shull, 1962 ; Stekol, Bedrak, Mody, Burnette and Somerville, 1963) and also NAD, the capacity for synthesis of which was reduced (Stekol *et al.*, 1963). However, a report cited by Reid (1962*a*) of unchanged NAD in rats fed thioacetamide for a prolonged period accords with the present observation of unchanged NAD in precancerous liver.

Reid and Morris (1963) have summarized the literature on nucleotides in transplanted hepatomas and have given data for a " minimum-deviation " hepatoma (" Morris 5123 "). The latter resembled the azo-dye hepatomas now studied in having decreased levels of purine nucleotides (including IMP) and of pyridine nucleotides other than NAD, but differed in having decreased levels of certain other nucleotides (such as UDP and UDPacetylglucosamine) which were usually increased or normal in the azo-dye hepatomas. If Potter's (1962) view be adopted, that a biochemical change cannot be considered as requisite for neoplasia unless it is found in all types of hepatoma examined, and if account be taken of data published for ascitic hepatomas (e.g. Mandel, Wintzerith, Klein-Pete and Mandel, 1963), then the " minimum deviations " in nucleotide levels that hepatomas must possess would appear to be decreases, perhaps moderate, in UDPglucuronate and $NADPH_2$. The present data for mouse hepatomas argue against the importance of the decreases in UDPglucose and NADP in the rat hepatomas. If data for ascitic hepatomas are disregarded on the grounds that comparison with normal liver may not be valid, the tendency towards depletion of purine nucleotides (notably IMP) in solid hepatomas might also be regarded as a " minimum deviation ". It is striking that in respect of most nucleotides the azo-dye hepatomas were, despite their fast growth rate, usually less abnormal than the Morris 5123 hepatomas.

*Relevance to hepatocarcinogenesis of the findings for ribonucleases and phosphodiesterases.*—The increase in the acid-ribonuclease activity of the supernatant fraction, as now found soon after commencement of feeding with 3′-Me-DAB, accords with other work (cited by Reid, 1962*a*) on precancerous liver and is evidently correlated with carcinogenicity ; as already indicated, the findings of Amano and Daoust (1961) may be irrelevant to the present findings. It appears, however, from preliminary experiments not reported here that a rise in this activity is not an early event in carcinogenesis by ethionine. Increased activity was also found in nodules induced by 3′-Me-DAB, except for some " small-celled " carcinomas. The supernatant-fraction activity (as distinct from the activity bound in lysosomes) may represent the activity actually available to the cell (Reid and Nodes, 1959), and an increase in this activity may imply faster catabolism of RNA, as reflected in the fall in microsomal RNA (Reid, 1964*a*). The finding that activity is likewise high in Morris 5123 hepatomas (Reid and Morris, 1963) suggests that this increase may rank as a " minimum deviation "; there is evidence that the increase is not an *in vitro* artefact (Reid and Nodes, 1963). One result of faster catabolism of RNA would be to make more uridine available for the synthesis of 5′-UMP (cf. Fig. 1).

The phosphodiesterases which open up the phosphate bridge in the cyclic monucleotides produced by ribonuclease action show high activity in normal liver ; moreover, cyclic mononucleotides have not been detected in liver. It is therefore unlikely that these phosphodiesterases are rate-limiting in RNA catabolism. Since, however, none of these enzymes is confined to a single sub-cellular fraction, it is conceivable that the enzyme in a particular fraction may have some special function. In each instance where activity towards a cyclic mononucleotide was somewhat altered in precancerous liver or hepatomas, the alteration was found in a sub-cellular fraction which is feebly active in normal rats—the microsomal fraction in the case of 2′,3′-AMP and 2′,3′-UMP, and the mitochondrial fraction in the case of 2′,3′-CMP. At present, however, no interpretation can be given of the observed changes in activity towards these substrates.

### SUMMARY

The levels of acid-soluble 5′-nucleotides and of DNA, and the activities of certain catabolic enzymes, have been determined in liver from rats fed carcinogenic or virtually non-carcinogenic azo dyes for several weeks, and in nodules (mostly hepatomas, of varying histological appearance) induced by prolonged feeding of 4-dimethylamino-3′-methylazobenzene (3′-Me-DAB). " Necrosis " as seen in some of the nodules had little influence on the biochemical pattern, and " hyperplastic nodules " resembled carcinomas. Nucleotides were also determined in liver from adrenalectomized or thyroxine-treated rats fed 3′-Me-DAB, and in hepatomas arising spontaneously in CBA mice. All results are expressed per g. of tissue.

In agreement with the literature, the activities of glucose-6-phosphatase and glucose-6-phosphate dehydrogenase were respectively decreased and increased in the dye-induced nodules. The latter activity also showed an eventual rise in precancerous liver. DNA was high in nodules and sometimes high—contrary to expectation—in early-precancerous liver.

Early during azo-dye feeding, uridine nucleotides showed a rise which, in the case of UMP and UTP, was apparently an important step in azo-dye carcinogenesis. In the nodules the levels of uridine nucleotides were often normal ; but UDP was somewhat high, and in fibrotic nodules UTP, UDPglucose, UDPglurcuronate and UDPacetylglucosamine were rather low. Purine nucleotides tended to be low in precancerous liver and especially in the nodules. The decreases in ADP, GDP and IMP were particularly consistent and were, for precancerous liver, correlated with the carcinogenicity of the dye. " Pyridine nucleotides " tended to diminish, but NAD was undiminished in precancerous liver and in some of the nodules ; the early decreases in NADP and $NADPH_2$ were well correlated with carcinogenicity. Of the changes now found in the nodules, only the decreases in UDP glucuronate and $NADPH_2$ and possibly those in certain purine nucleotides are likely to rank as " minimum deviations ", as judged by results for the mouse hepatomas and by published evidence.

The acid-ribonuclease activity of supernatant fractions showed, even with one week of 3′-Me-DAB feeding, a marked rise which may well be an important step in azo-dye carcinogenesis. Increased activity was also seen in the nodules other than some " small-celled " carcinomas. Mitochondrial or microsomal fractions from precancerous liver and nodules showed altered phosphodiesterase activity towards certain nucleoside cyclic phosphates (cyclic mononucleotides).

We are grateful to Dr. S. Doak for giving numerous histological opinions, to Miss P.M. Law and Mr. C. K. Grant for technical help, to Mr. C. Smith for help with maintenance of the animals, to Dr. D. I. Connell for providing the mouse hepatomas, and to Mr. K. G. Moreman for the photomicrographs. The work was supported by grants to the Chester Beatty Research Institute (Institute of Cancer Research : Royal Cancer Hospital) from the Medical Research Council, the British Empire Cancer Campaign, the Anna Fuller Fund, and the National Cancer Institute of the National Institutes of Health, U.S. Public Health Service.

### REFERENCES

Amano, H. and Daoust, R.—(1961) *J. Histochem. Cytochem*, **9**, 161.
Beaufay, H., Van Campenhout, E. and De Duve, C.—(1959) *Biochem. J.*, **73**, 617.
Brown, D. M., Magrath, D. I. and Todd, A. R.—(1952) *J. chem. Soc.*, 2708.
Burton, K.—(1956) *Biochem. J.*, **62**, 315.
Caiger, P., Morton, R. K., Filsell, O. H. and Jarrett, I. G.—(1962) *Ibid.*, **85**, 351.
Caldarera, C. M., Budini, R., Barbiroli, B. and Rabbi, A.—(1962) *Cancer Res.*, **22**, 1026.
Chayen, J., Bitensky, L., Aves, E. K., Jones, G. R. N., Silcox, A. A. and Cunningham, G. J.—(1962) *Nature, Lond.*, **195**, 715.
Connell, D. I. and Alexander, P.—(1959) *Gerontologia*, **3**, 153.
Cunningham, L., Griffin, A. L. and Luck, J. M.—(1950) *J. gen. Physiol.*, **34**, 59.
Emmelot, P., Bos, C. J., Brombacher, P. J. and Hampe, J. F.—(1959) *Brit. J. Cancer*, **13**, 348.
Forrest, R. J., Wilkin, D. R. and Hansen, R. G.—(1960) *Biochim. biophys. Acta*, **37**, 551.
Glock, G. E. and McLean, P.—(1955) *Biochem. J.*, **61**, 397.—(1957) *Ibid.*, **65**, 413.
Goldacre, R. J. and Sylvén, B.—(1962) *Brit. J. Cancer*, **16**, 306.
Griffin, A. C., Nye, W. N., Noda, L. and Luck, J. M.—(1948) *J. biol. Chem.*, **176**, 1225.
Hadjiolov, A. A.—(1962) *Bull. Soc. Chim. biol., Paris*, **44**, 745.
Henderson, J. F.—(1962) *J. biol. Chem.*, **237**, 2631.
Hommes, F. A., Leeuwen, G. Van, and Zilliken, F.—(1962) *Biochim. biophys. Acta*, **56**, 320.
Iversen, S. and Thamsen, A.—(1956) *Acta path. microbiol. scand.*, **38**, 96.
Jedeikin, L., Thomas, A. J. and Weinhouse, S.—(1956) *Cancer Res.*, **16**, 867.
Knox, W. E., Auerbach, V. H. and Lin, E. C. C.—(1956) *Physiol. Rev.*, **36**, 164.
Kotnis, L. B., Narurkar, M. V. and Sahasrabudhe, M. B.—(1962*a*) *Brit. J. Cancer*, **16**, 541.—(1962*b*) *Ibid.*, **16**, 550.
Ledoux, L., Pileri, A. and Vanderhage, F.—(1958) *Rev. franc. Étud. clin. biol.*, **7**, 776.
Magasanik, B. and Karibian, D.—(1960) *J. biol. Chem.*, **235**, 2672.
Maini, M. M. and Stich, H. F.—(1961) *J. nat. Cancer Inst.*, **26**, 1413.
Mandel, P., Wintzerith, M., Klein-Pete, N. and Mandel, L.—(1963) *Nature, Lond.*, **198**, 1000.

MONDY, N. I., STRENGTH, D. R., GRAY, L. F. AND DANIEL, L. J.—(1954) *Proc. Soc. exp. Biol. N.Y.*, **87**, 129.
MORRIS, H. P.—(1963) *Progr. exp. Tumor Res.*, **3**, 370.
MORTON, R. K.—(1958) *Nature, Lond.*, **181**, 540.
NODES, J. T.—(1958) *Biochim. biophys. Acta*, **32**, 551.
*Idem* AND REID, E.—(1962) *Biochem. J.*, **83**, 4P.
*Iidem* AND WHITCUTT, J. M.—(1962) *Ibid.*, **84**, 33P.
PITOT, H. C.—(1960) *Cancer Res.*, **20**, 1262.—(1962) *Fed. Proc.*, **21**, 1124.
POTTER, V. R.—(1962) " The Molecular Basis of Neoplasia ", University of Texas, M. D. Anderson Hospital and Tumor Institute. Austin (Univ. of Texas Press) p. 367.
PRICE, J. M., HARMAN, J. W., MILLER, E. C. AND MILLER, J. A.—(1952) *Cancer Res.*, **12**, 192.
*Idem,* MILLER, E. C., MILLER, J. A. AND WEBER, G.—(1950) *Ibid.*, **10**, 18.
REID, E.—(1958) *Brit. J. Cancer*, **12**, 428.—(1961) *Mem. Soc. Endocrin.*, **11**, 149.—(1962*a*) *Cancer Res.*, **22**, 398.—(1962*b*) *Nature, Lond.*, **194**, 1153.—(1964*a*) *Brit. J. Cancer*, in press.—(1964*b*) *Ibid.*, in press.
*Idem* AND LOTZ, F.—(1958) *Ibid.*, **12**, 419.
*Idem* AND MORRIS, H. P.—(1963) *Biochim. biophys. Acta*, **68**, 647.
*Idem* AND NODES, J. T.—(1959) *Ann. N.Y. Acad. Sci.*, **81**, 618.—(1963) *Nature, Lond.*, **199**, 176.
REYNAFARJE, B. AND POTTER, V. R.—(1957) *Cancer Res.*, **17**, 1112.
SCHMITZ, H.—(1961) 11 Colloq. der Gesellschaft für Physiol. Chimie. Berlin (Springer-Verlag), p. 1.
SHULL, K. H —(1962) *J. biol. Chem.*, **237**, PC1734.
STEKOL, J. A., BEDRAK, E., MODY, U., BURNETTE, N. AND SOMERVILLE, C.—(1963) *Ibid.*, **238**, 469.
STRIEBICH, M. J., SHELTON, E. AND SCHNEIDER, W. C.—(1953) *Cancer Res.*, **13**, 279.
WADA, T., OHARA, H., SASAKI, T., NAKAJIMA, J. AND YACHI, A.—(1957) *Gann*, **48**, 305.
WRBA, H., SCHÖNENBERGER, H., BAMANN, E., AND LANG, R.—(1961) *Naturwissenschaften*, **48**, 625.
WYNGAARDEN, J. B. AND ASHTON, D. M.—(1959) *J. biol. Chem.*, **234**, 1492.